# The long and short of it: benchmarking viromics using Illumina, Nanopore and PacBio sequencing technologies

**DOI:** 10.1099/mgen.0.001198

**Published:** 2024-02-20

**Authors:** Ryan Cook, Nathan Brown, Branko Rihtman, Slawomir Michniewski, Tamsin Redgwell, Martha Clokie, Dov J. Stekel, Yin Chen, David J. Scanlan, Jon L. Hobman, Andrew Nelson, Michael A. Jones, Darren Smith, Andrew Millard

**Affiliations:** ^1^​ School of Veterinary Medicine and Science, University of Nottingham, Sutton Bonington Campus, College Road, Loughborough, Leicestershire, LE12 5RD, UK; ^2^​ Centre for Phage Research, Dept Genetics and Genome Biology, University of Leicester, University Road, Leicester, Leicestershire, LE1 7RH, UK; ^3^​ School of Life Sciences, University of Warwick, Gibbet Hill Road, Coventry, CV4 7AL, UK; ^4^​ Warwick Medical School, University of Warwick, Gibbet Hill Road, Coventry, CV4 7AL, UK; ^5^​ COPSAC, Copenhagen Prospective Studies on Asthma in Childhood, Herlev and Gentofte Hospital, University of Copenhagen, Ledreborg Alle 34, 2820, Gentofte, Denmark; ^6^​ School of Biosciences, University of Nottingham, Sutton Bonington Campus, College Road, Loughborough, Leicestershire, LE12 5RD, UK; ^7^​ Department of Mathematics and Applied Mathematics, University of Johannesburg, Rossmore 2029, South Africa; ^8^​ Faculty of Health and Life Sciences, University of Northumbria, Newcastle upon Tyne, NE1 8ST, UK

**Keywords:** bacteriophage, bioinformatics, hybrid assembly, Illumina, long-reads, Nanopore, PacBio, Phage, viral metagenomics, Viromics

## Abstract

Viral metagenomics has fuelled a rapid change in our understanding of global viral diversity and ecology. Long-read sequencing and hybrid assembly approaches that combine long- and short-read technologies are now being widely implemented in bacterial genomics and metagenomics. However, the use of long-read sequencing to investigate viral communities is still in its infancy. While Nanopore and PacBio technologies have been applied to viral metagenomics, it is not known to what extent different technologies will impact the reconstruction of the viral community. Thus, we constructed a mock bacteriophage community of previously sequenced phage genomes and sequenced them using Illumina, Nanopore and PacBio sequencing technologies and tested a number of different assembly approaches. When using a single sequencing technology, Illumina assemblies were the best at recovering phage genomes. Nanopore- and PacBio-only assemblies performed poorly in comparison to Illumina in both genome recovery and error rates, which both varied with the assembler used. The best Nanopore assembly had errors that manifested as SNPs and INDELs at frequencies 41 and 157 % higher than found in Illumina only assemblies, respectively. While the best PacBio assemblies had SNPs at frequencies 12 and 78 % higher than found in Illumina-only assemblies, respectively. Despite high-read coverage, long-read-only assemblies recovered a maximum of one complete genome from any assembly, unless reads were down-sampled prior to assembly. Overall the best approach was assembly by a combination of Illumina and Nanopore reads, which reduced error rates to levels comparable with short-read-only assemblies. When using a single technology, Illumina only was the best approach. The differences in genome recovery and error rates between technology and assembler had downstream impacts on gene prediction, viral prediction, and subsequent estimates of diversity within a sample. These findings will provide a starting point for others in the choice of reads and assembly algorithms for the analysis of viromes.

## Data Summary

All reads from virome sequencing were submitted to the ENA under study PRJEB56639. The assemblies are provided via FigShare (https://doi.org/10.25392/leicester.data.21346935.v2)[1].

**Impact Statement**

Viral metagenomics is used study bacteriophage diversity and ecology. While long-read sequencing has been widely used in bacterial genomics and metagenomics, its use in viral communities is still in its early stages. The work provides the first comparison of utilizing Illumina, Oxford Nanopore, and PacBio sequencing technologies to sequence a mock bacteriophage community. Highlighting the choice of assembler and sequence technology can have an impact on the predicted diversity of the resultant viromes.

## Introduction

Viruses play critical roles in all environments they inhabit, as evidenced by their distribution and abundance. In particular, viruses that infect bacteria, bacteriophages (from hereon phages), are known to play important roles in regulating the abundance of their bacterial hosts, facilitating horizontal gene transfer, and playing crucial roles in global biogeochemical cycles by augmenting host metabolism [2–4].

It is now over 40 years since sequencing of the first phage genome [5]. The number of complete phage genomes from phage isolates is now >22 000 [6]. However, millions more phage genomes have been sequenced through metagenomic sequencing and are available through a variety of databases [7–9]. Viral metagenomics (viromics) has revolutionized our understanding of the diversity of phages and their potential ability to augment host metabolism. Initial virome studies required DNA to be cloned into a vector and the clone sequenced by Sanger sequencing. As new sequencing technologies developed that did not require the cloning of DNA, such as Solexa (becoming Illumina), 454, and SOLiD, the field of viromics expanded. With Illumina sequencing becoming the dominant technology, more and more viromes have been sequenced spanning pristine ocean environments [10], abyssal depths [11], and even the faeces of a wide variety of animal species [12–14].

Whilst viromes produced using Illumina short-read sequencing have provided new insights into viral diversity, short-reads are not able to resolve all viral genomes within a virome. Phages that contain hypervariable regions and/or possess high microdiversity are known to cause virome assemblies to fragment, resulting in reduced contig sizes and exclusion from further analyses [15]. To overcome such problems, alternative approaches to viromics can be taken including single-cell viromics or the cloning of viral genomes into fosmids [16]. Whilst both of these approaches are beneficial, they are technically challenging compared to more standard viromics workflows.

Recent technological developments have led to the production of long-read sequences by both Oxford Nanopore Technology (ONT) [17] and PacBio [18]. While the technologies differ in their approach, both platforms sequence single molecules and are capable of producing sequences of tens of kilobases in length [18]. The ability to sequence long DNA molecules offers the ability to overcome the issues of microdiversity and/or hypervariable regions found within phage genomes [15]. To date there have been limited studies using ONT sequencing for viromics. One of the first was able to acquire complete phage genomes from single ONT reads, utilizing tangential flow filtration (TFF) of marine samples to obtain the significant amounts of DNA required for library preparation [19]. Extraction of such quantities of phage DNA is likely prohibitive from more viscous and heterogeneous environments where multiple displacement amplification (MDA) is already used to obtain enough DNA for library preparation for short-read sequencing. While MDA provides a solution to the amount of input material, it does not come without problems. It has been well documented that MDA can introduce biases in metagenomic libraries, in particular over representation of ssDNA phages within samples [20–22]. To overcome the problem of library input requirements, MDA for ONT library preparation, combined with unamplified short-read libraries for quantification have been utilized [23]. Alternatively, ONT sequencing (MinION) of long-read linker amplified shotgun libraries (LASL), to sequence PCR products on a MinION, combined with Illumina short-reads were used in an approach dubbed virION [15, 24]. Both approaches were successful in increasing the number and completeness of viral genomes.

While the number of viromes that utilize ONT alone or in combination with Illumina sequencing is slowly increasing [15, 23–26], reports of utilizing PacBio sequencing for viromes are scarce [27]. A recent study predicted phages from a bacterial metagenome assembled from PacBio reads, identifying phages not identified when the same sample was sequenced with short-reads [27]. It is not clear why there are not more viromes sequenced with long-read technologies, as has become commonplace for sequencing of bacterial metagenomes. Even for the sequencing of individual phage isolates there are relatively few studies that have utilized long-reads [28–32]. In part, this is likely because the vast majority of phage genomes can be assembled from short-read Illumina sequences alone [33]. Thus, unlike sequencing their bacterial hosts, long-reads do not provide the immediate benefit of a better genome assembly for an isolate and thus the need to use them is reduced. The lack of long-read data generally for phage isolates, combined with the lack of a comparative benchmarked dataset comparing different methods is likely contributing to long-read sequencing not being widely adopted for viromes, despite clear benefits from the limited studies performed to date. We aimed to understand how different sequencing technologies affect the recovery of viral genomes from communities and the quality of the assembled genomes.

Here we sequenced a mock community of phages with three different sequencing technologies (PacBio, MinION, and Illumina) in order to benchmark these different approaches and identify the benefits and limitations of each approach. To do this we utilized five different assembly algorithms: SPades, Unicyler, Flye, Wtdgb2, and Canu. Furthermore, we utilized the virus prediction software VIBRANT and DeepVirFinder to identify viral contigs from contaminating host DNA, to assess the effect of sequencing technology on predicted community diversity.

## Methods

### Mock virome community preparation and sequencing

Phages (vB_Eco_SLUR29, vB_EcoS_swan01 [34], vB_Eco_mar001J1 [34], vB_Eco_mar002J2 [34], KUW1 (OQ376857), PARMAL1 (OQ376857), HP1 [33], DSS3_PM1, vB_Eco_mar005P1 [34], S-RSM4 [35], vB_Eco_mar003J3 [34], vB_Vpa_sm033,vB_VpaS_sm032, CDMH1 [33]) were propagated as previously described and DNA was extracted using the method of Rihtman *et al.* [33]. DNA was quantified with the Qubit dsDNA high-sensitivity kit. ΦX174 DNA was obtained from the spike in control provided with Illumina library preparation kits. Genomic DNA was combined to produce a mock community of 15 phages that covered a range of lengths (44 509–320 253 bp) and molGC content (38 –61 %). Genomes were combined across a range of abundances (169 000–684 329 545 genome copies) within the mock community (Table S1, available in the online version of this article). Genome copies were estimated by using the formula: (ng of DNA * 6.022×10^23^) / (genome length * 660 * 1×10^9^). The genomes were chosen to include both highly divergent and highly similar phages (Table S1, Fig. S1).

Illumina library preparation was carried out using the NexteraXT library preparation kit, with a minor modification to the number of PCR cycles as described previously [34]. In addition, no ΦX174 spike was added to the library as it is part of the normal Illumina library preparation protocol. Sequencing was carried out with a MiSeq 2×250 bp kit. For MinION and PacBio sequencing, the DNA was amplified prior to sequencing with the GenomiPhi V3 DNA Amplification Kit, following the manufacturer’s instructions. Eight individual amplification reactions were performed with 10 ng DNA input for each amplification. Following amplification, DNA was treated with S1 nuclease with 10 U per μg of input DNA and the enzyme deactivated, prior to cleanup and concentration with a DNA Clean and Concentrator-25 column (Zymo Research). Three independent amplification reactions were sequenced via PacBio or ONT sequencing.

Libraries were prepared for MinION sequencing using SQK-LSK109 (Version: NBE_9065_v109_revB_23May2018) with the native barcoding kit, following the manufacturer’s instructions (Oxford Nanopore Technologies, Oxford, UK) with omission of the initial g-tube fragmentation step. Base calling was carried out with Guppy v6.4.6 using ‘dna_r9.4.1_e8.1_hac.cfg’, with reads demultiplexed using Porechop [36]. PacBio sequencing was carried out at NU-OMICS (Northumbria University). Briefly, genomic DNA was sheared using g-TUBE (Covaris, USA) to an average size of 8–10 kb and then libraries were prepared using SMRTbell Template Prep Kit 1.0 (Pacific BioSciences, USA) as per the manufacturer’s instructions and sequenced on the Sequel I system (Pacific BioSciences, USA). Circular consensus reads were created in SMRTLink v6 (Pacific BioSciences, USA) and fastq files were generated using the BAM to FASTX pipeline.

### Bioinformatics analyses

Read library sizes were determined using the ‘stats’ command as part of SeqFu v1.20.0 [37]. To determine coverage and depth, reads from each library were mapped to the 15 reference genomes using Minimap2 v2.14-r892-dirty with ‘-axe sr’, ‘-axe map-ont’ or ‘-axe map-pb’ for Illumina, ONT and PacBio reads, respectively [38]. Minimap2 output was piped and sorted using the Samtools sort command to produce sorted bam files [39]. Coverage and depth were taken from the bam files using the Samtools coverage command [39].

Assemblies were separately produced for the three libraries, and additional assemblies were produced by pooling the three libraries together, resulting in four assemblies per read/assembler combination. Following assembly contigs were ‘polished’ with long- or short-reads, polishing is the process of removing residual small-scale errors, by the mapping of reads back against the assembly to identify and correct such errors. Illumina reads were trimmed with Trim Galore v0.4.3 with default parameters prior to assembly [40]. Illumina reads were assembled using SPAdes v3.12.0 with parameters ‘--meta -t 16’ [41]. ONT reads were quality controlled with Filtlong v0.2.1 with the parameters ‘--min_length 1000 --keep_percent 90’. Flye v2.9.1 assemblies were produced with parameters ‘ --nano-hq -t 40’ or ‘--pacbio-raw’ [42]. Unicycler v0.5.0 assembly of long-reads was used with ‘--linear 14’ [43], that utilizes miniasm [44] for an overlay consensus assembly followed by racon for polishing [45]. wtdbg2 v2.5 was used with the parameters ‘-p 21 k 0 -AS 4 K 0.05 s 0.05 L 1000 --edge-min 2 --rescue-low-cov-edges -t 90’ [46]. Long-reads were also assembled with Canu v2.2 using the following parameters ‘canu -correct -nanopore –genomeSize=800 k, ‘canu -trim -corrected genomeSize=800 k -nanopore’, ‘canu -trimmed -corrected genomeSize=800 k -nanopore’. Long-read ONT assemblies were polished using medaka v1.7.2 with the following settings ‘medaka_consensus -m r941_min_hac_g507 -t 40’, with four rounds of polishing.

To determine whether using long- and short-reads together improved the assemblies, three methods that utilized a hybrid approach were used. (1) Long-read-only assemblies underwent one round of polishing with Polypolish v0.5.0 [47], as suggested in the Polypolish documentation (https://github.com/rrwick/Polypolish). Firstly, reads from respective Illumina libraries were mapped using bwa mem v0.7.17-r1188 [48]. Alignments were filtered using polypolish_insert_filter.py, and Polypolish was then used with default parameters [47]. These assemblies are hereafter referred to as ‘polished’ (2). For a hybrid assembly with Unicycler, long- and short-reads were provided with default parameters (hereafter referred to as ‘hybrid’). (3) The Flye assemblies polished with Illumina reads were combined with the Illumina-only assemblies and de-replicated using MIUViG [49] recommended thresholds [95 % average nucleotide identity (ANI) over 85 % genome length] using blast v2.13.0 alongside the anicalc and aniclust scripts available as part of CheckV (https://bitbucket.org/berkeleylab/checkv/src/master/) [50], similar to a previously performed analysis of agricultural viromes [23].

To assess completeness and quality, assemblies were compared to the 15 reference genomes using metaQUAST v5.0.2 with default parameters [51]. All resultant plots were produced using ggplot2 in R v3.5.1. When investigating the fidelity of assemblies to the reference genomes, we included assemblies for which 50 % of the genome was covered by contigs, no matter how fragmented the assembly was (i.e. if 100 individual contigs mapped to 50 % of genome length, despite the longest contig only being 10 % of genome length, this was still included. This was to exclude misassembly data for which only small portions of genomes were assembled, potentially leading to under-estimation of error frequencies). To investigate the effect of sequence depth on long-read assembly, reads mapping to the genome of interest were extracted and sub-sampled using seqtk sample with ‘-s 100 frac’, with frac set to the desired depth.

To determine the effect of polishing long-read assemblies with short-reads on viral prediction software, we processed the long-read assemblies and their polished counterparts using vibrant v1.2.1 [52] with the following parameters ‘-t 8 l 10000 -virome’ and compared against DeepVirFinder v1.0 [53] with contigs >10 kb and a *P*-value<0.05. Prodigal v2.6.2 with default settings was used for predicting open reading frames on the viral operational taxonomic units (vOTUs) and the 15 reference genomes [54]. As in the norm in virome studies, we defined a viral operational taxonomic unit (vOTU) as any contig ≥10 kb in length as used by Roux *et al*. [55].

To investigate the effect of different sequencing platforms and assemblers on estimates of viral diversity, we applied a typical virome analysis workflow to the assemblies. Each assembly was separately processed using DeepVirFinder v1.0 [53]. Contigs ≥10 kb were included as a vOTU if they obtained a *P*-value of ≤0.05 from DeepVirFinder or were indicated as viral by vibrant. Reads from the corresponding Illumina library were mapped to the assembly using Bbmap v38.69 at 90 % minimum ID and the ambiguous=all flag [56]. vOTUs were deemed as present in a sample if they obtained ≥ 1×coverage across ≥ 75 % of contig length [55]. The number of reads mapped to present vOTUs were normalized to reads mapped per million. Relative abundance values were analysed using Phyloseq v1.26.1 [57] in R v3.5.1 to calculate diversity statistics [58]. The number of predicted vOTUs and alpha diversity statistics were compared to the genome copy numbers used in the original mock community. To complement the prediction of viral contigs, we identified the proportion of reads that mapped to host bacterial genomes using bbmap.sh v38.86 with the covstats options [56]. Bacterial genomes of the following accessions were used as reference: NC_009089.1, U00096.3, L42023.1, NC_003911.12, NC_004605.1,NC_004603.1.

## Results

### Mock virome composition

To assess the performance of short-, long- and hybrid-sequencing approaches for viromic analyses, we sequenced a mock community of 15 bacteriophage genomes with an Illumina MiSeq, PacBio Sequel, and ONT MinION. For Illumina sequencing, no MDA was used to provide a library as free as possible from bias. For PacBio and ONT sequencing, the mock community was first amplified with MDA to obtain sufficient material for library preparation and sequencing. The amplification of DNA is often required for the study of viruses in faecal matter, due to the limitations of obtaining enough sample [59]. Three independent samples were sequenced on each platform and the fourth sample created by pooling the three samples, to create one pseudo deep sequenced sample. The Illumina, ONT and PacBio libraries yielded 0.5–1.1 Gb, and 0.6–0.9 Gb, and 0.3–0.5 Gb of data, respectively. Pooling the libraries resulted in 2.4, 2.3 and 1.1 Gb for Illumina, ONT and PacBio libraries, respectively (Table S3).

### Limits of detection by read mapping

**Fig. 1.**
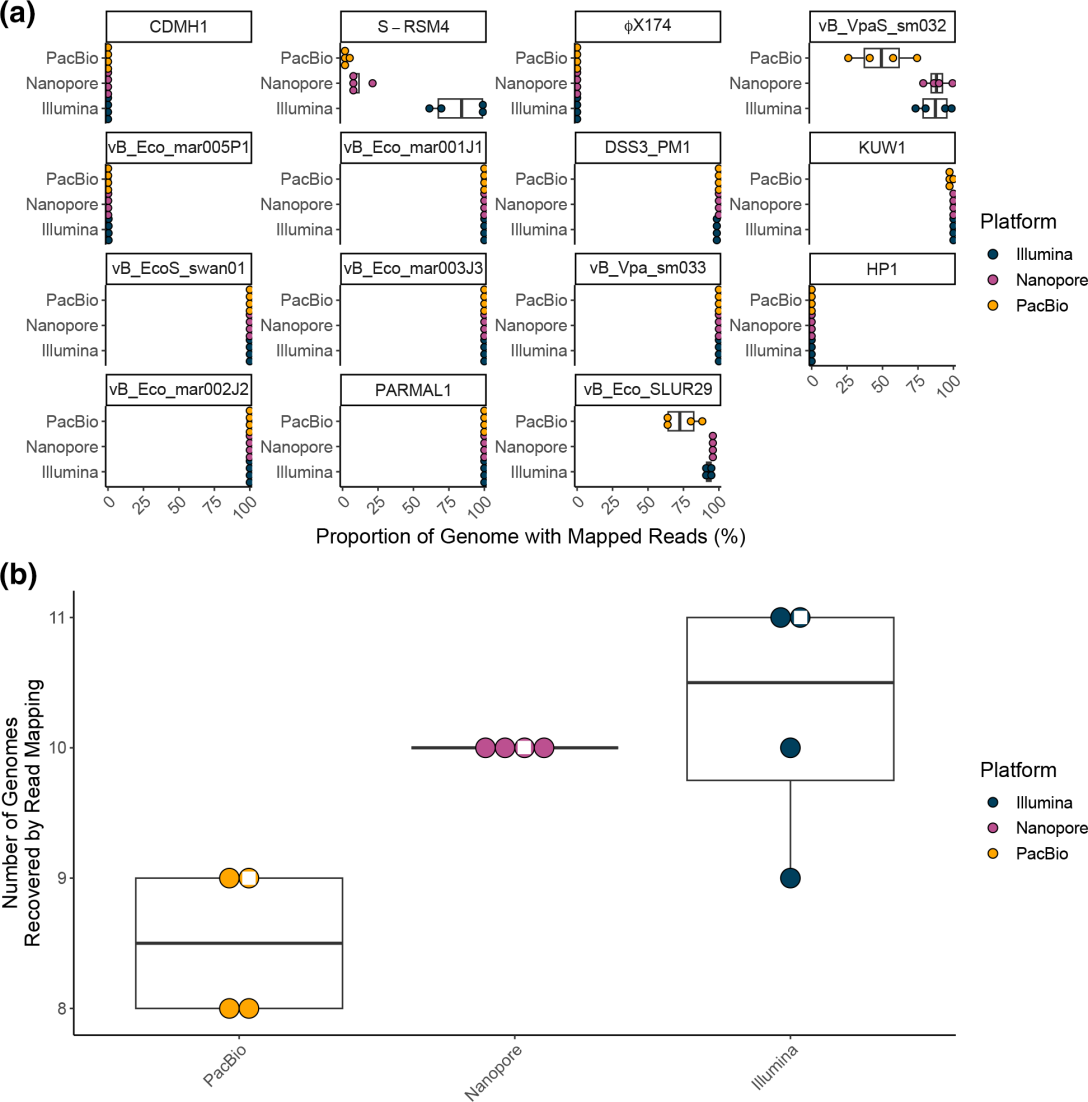
Detection of genomes by read mapping. (**a**) Dot and boxplots showing the proportion of each genome to which reads were mapped from each of the three sequencing platforms for library repeats with genomes ordered from lowest (CDMH1) to highest (vB_Eco_SLUR29) input quantities. (**b**) The number of genomes detected by read mapping by each sequencing platform at 1xcoverage over ≥75 % of genome length with the white diamond indicating the pooled library. The input mock community contained 15 genomes as an input. Three independent libraries were prepared and sequenced on each technology, these three independent libraries were then pooled to create the fourth deeply sequenced sample.

First, we assessed the limits of detection of each sequencing platform using a mapping-based approach, with detection of a genome set at 1×coverage across ≥75 % of a genome. Four phage genomes were not detected at all (CDMH1, HP1, vB_Eco_mar005P1 and ΦX174) by any sequencing technology (Fig. 1a). The Illumina libraries detected the highest number of genomes (9–11 genomes), with all ONT libraries detecting 10, and PacBio detecting 8–9 genomes (Fig. 1b). The least abundant phage to be detected was S-RSM4 (465 530 copies) and was only detected by Illumina sequencing, although a small percentage of the genome was covered in the PacBio and ONT libraries. The least abundant phages detected in ONT and PacBio libraries were vB_VpaS_sm032 (52 465 265 copies) and vB_Eco_mar001J1 (53 672 906 copies), respectively.

**Fig. 2.**
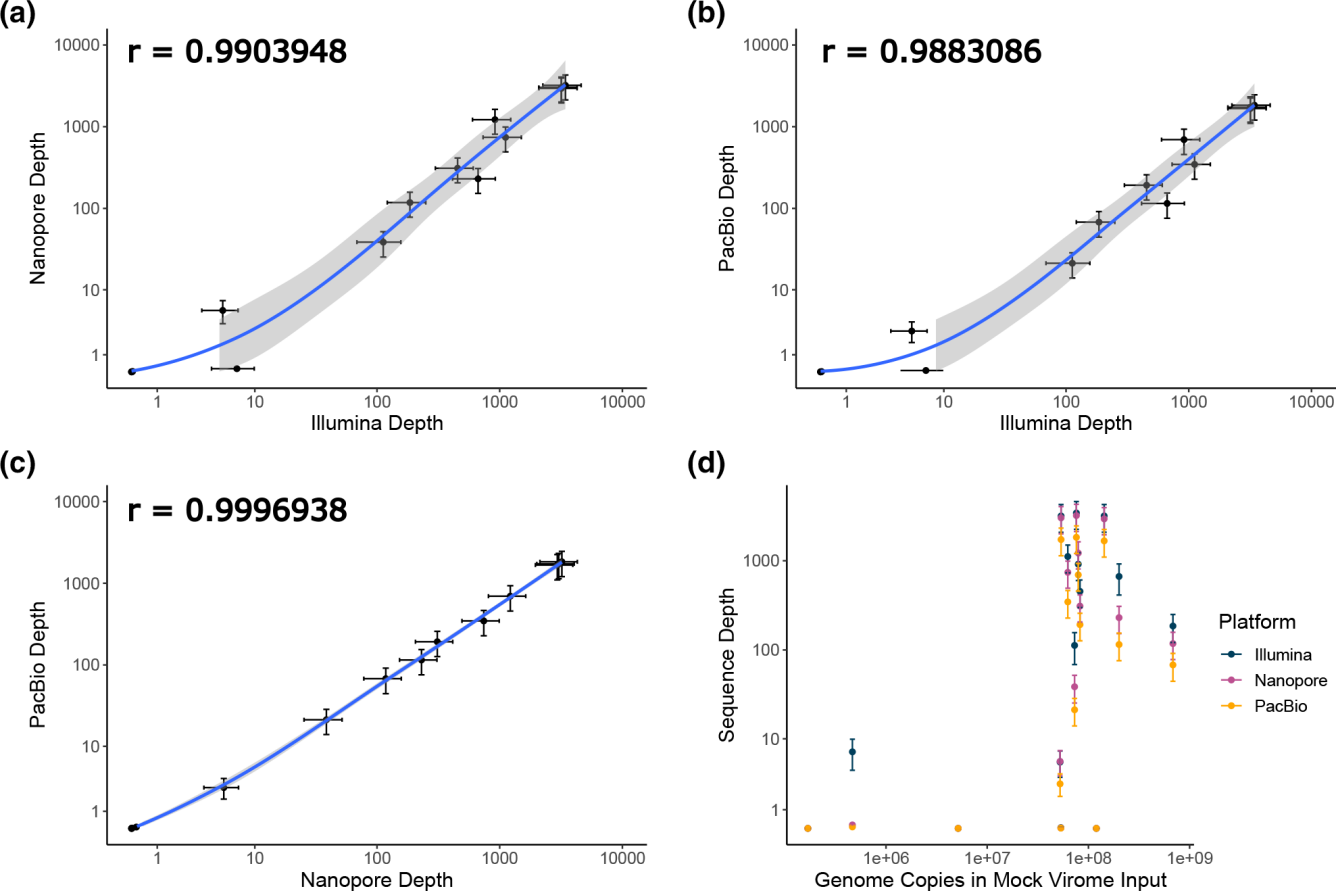
Comparison of sequencing depth between platforms. Correlation plots showing average sequence depth of a genome between (**a**) Illumina and ONT, (**b**) Illumina and PacBio, and (**c**) ONT and PacBio. An additional plot (**d**) shows sequence depth for the three sequencing platforms versus the estimated number of genome copies in the original mock community from which DNA libraries were prepared and sequenced. Values shown are the mean across three libraries and a pooled library, with bars showing standard error.

The use of unamplified DNA for Illumina libraries allowed any effects of MDA to be identified in the long-read assemblies. Encouragingly, the abundance of a genome within a sample generally correlated across different sequencing platforms, even after MDA for PacBio and ONT sequencing (ONT vs Illumina r=0.99, PacBio vs Illumina r=0.99, ONT vs PacBio r=0.99) (Fig. 2a, b and c; Table S4). However, it should be noted that phage ΦX174 was not detected in any sample, suggesting we may have been overly cautious in the amount we added to the mock community.

### Assembly results – genome recovery

Whilst known viruses can be detected by mapping, given the vast diversity of viruses in different environments, it is essential to be able to assemble viruses from a sample to identify viruses not currently in databases. Therefore, we tested the ability to assemble genomes from a sample. As assembly options for each read type were tested to optimize assembly methods, assemblies were obtained for all samples and assemblers tested, with the exception of PacBio reads using Unicycler (miniasm+racon) so these were excluded from further analysis. To investigate whether combining read technologies led to more complete assemblies, PacBio and ONT reads were separately assembled alongside Illumina reads using Unicycler to produce ‘hybrid’ assemblies. The Flye assemblies polished with Illumina reads were separately combined with Illumina-only assemblies and de-replicated at 95 % average nucleotide identity (ANI) over 85 % length to produce ‘deduped’ assemblies [50].

**Fig. 3.**
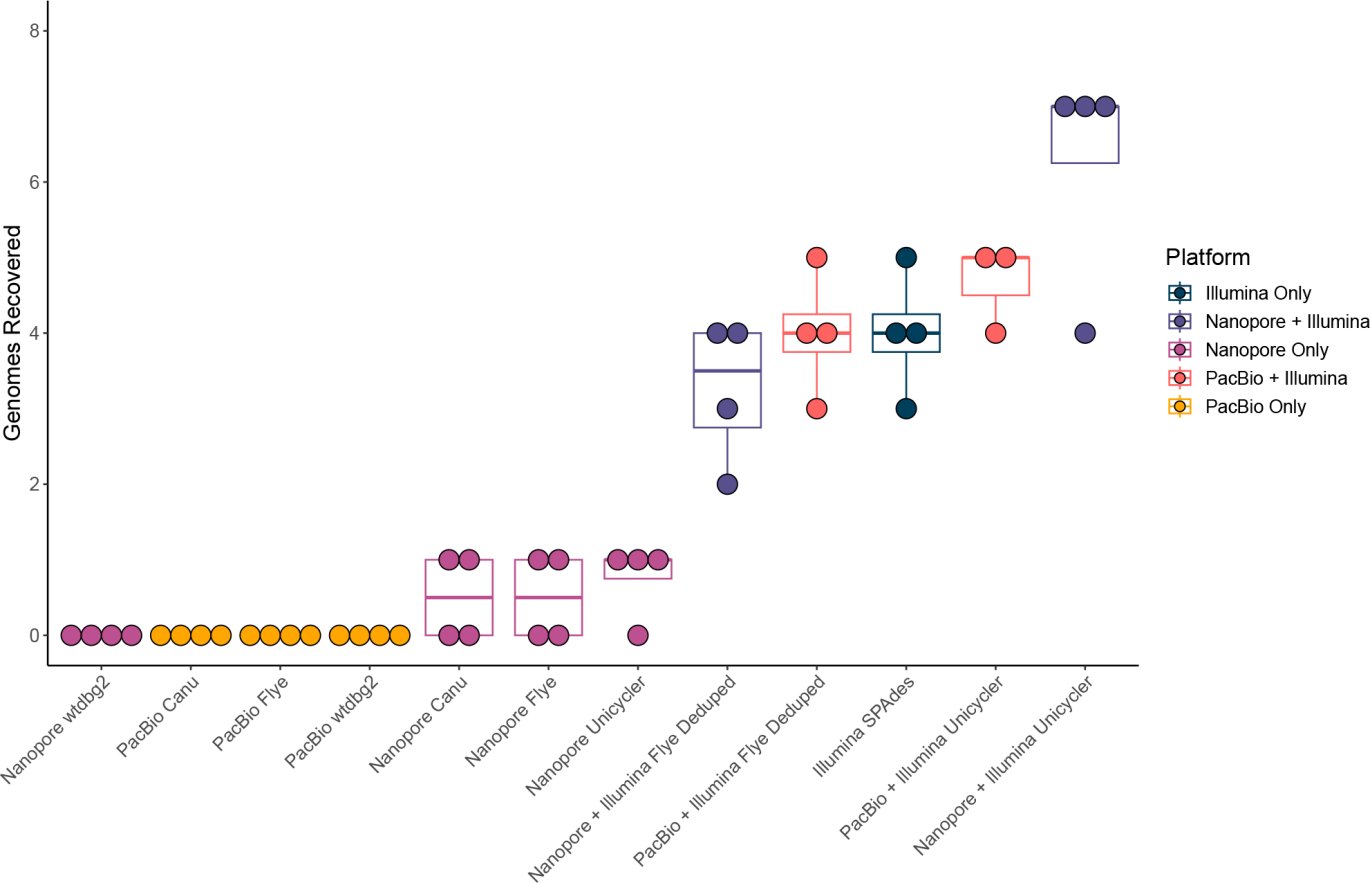
Comparison of genome recovery across sequencing technologies and assemblies. The mock community contained 15 genomes as an input. Dot and boxplot showing the number of genomes fully assembled within each assembly (successful assembly defined as a single contig covering 97 % of genome length), with the reads used for assembly shown in different colours.

**Fig. 4.**
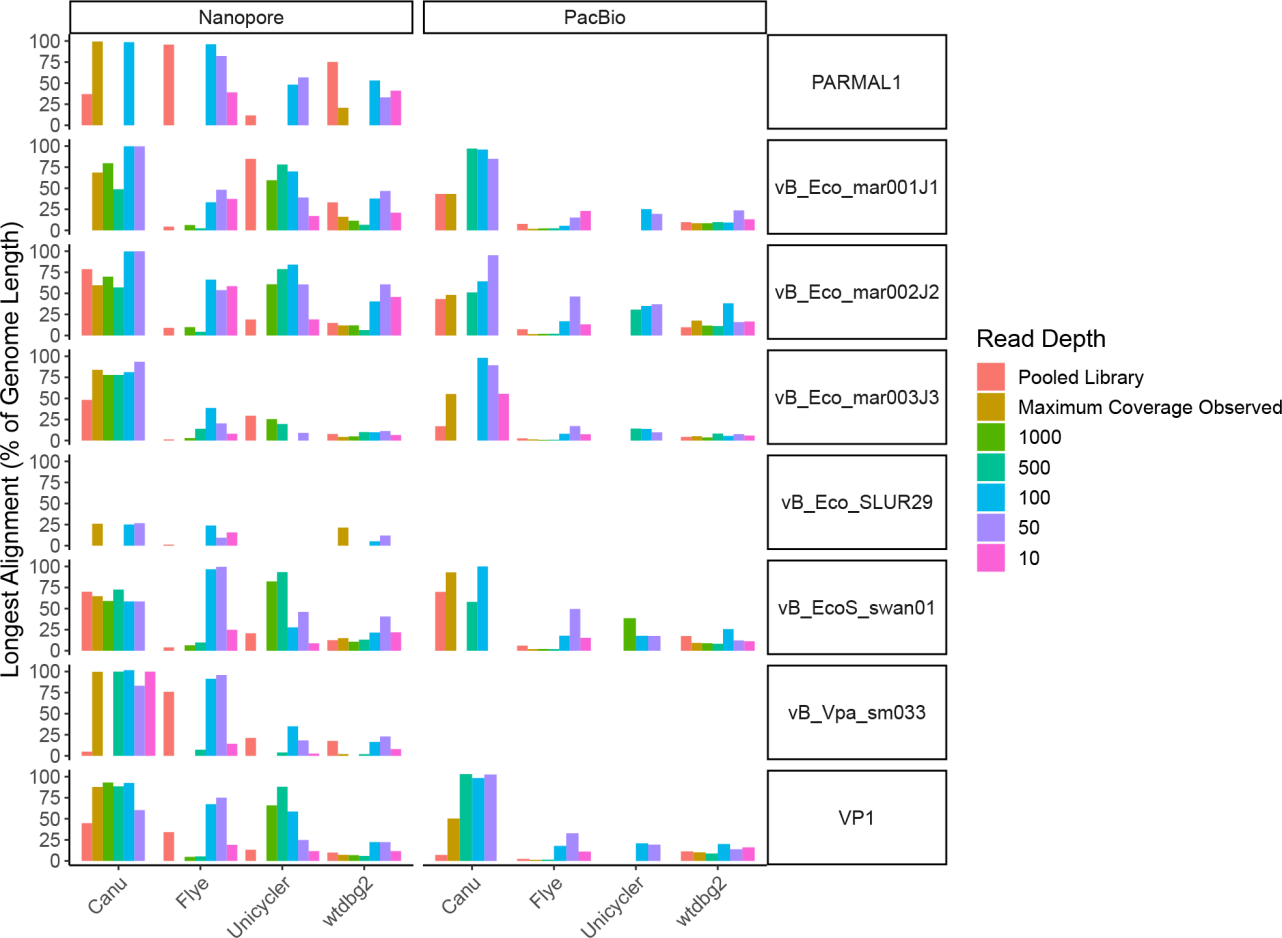
Effect of read depth on long-read assembly. To determine if read depth was a factor in genome assembly when using long-reads. Reads were first mapped to individual genomes, then randomly subsampled from the maximum coverage obtained in a sample, down to give 1000x, 500x, 100x, 50x and 10x coverage per genome, before being assembled. The proportion of the original reference genome (%) that was assembled into a single contig was then determined.

For individual sequencing platforms, using only short-reads (Illumina) produced the highest number of completely assembled genomes (3–5) (Figs 3 and S2). Using ONT a single genome was assembled with Canu, Flye and Unicycler, and none with wtdbg2. The PacBio assemblies obtained no complete genomes. Despite having >500×coverage of some genomes in long-read-only libraries, the reads did not assemble into complete genomes, suggesting high coverage may be a hindrance to assembly in some cases (Table S4, Fig. 4). The Illumina+ONT hybrid assembly (Unicycler) recovered the most genomes (4–7 genomes), and the Illumina+PacBio hybrid (Unicycler) assembly recovered 4–5 genomes. (Figs 3 and S2). Thus, the addition of long-reads to short-reads increased the number of genomes recovered (particularly ONT).

### Assembly results – limits of detection for assembled genomes

The phage with the lowest input abundance to be recovered in a single contig within any assembly was vB_Eco_mar001J1 (53 672 905 genome copies), which was recovered in the ONT+Illumina hybrid assembly. The least abundant genome to be recovered from an Illumina only assembly was KUW1 (72 995 151 genome copies), which was also the least abundant genome detected in a PacBio+Illumina hybrid assembly. Whilst not the genome with lowest input abundance, KUW1 was recovered from Illumina libraries at a lower average sequence depth than any other genome (139×coverage in the largest Illumina library, 225×coverage in the pooled library). Furthermore, KUW1 was not assembled in the two smaller Illumina libraries (37 and 49×coverage obtained), suggesting that the depth of Illumina sequencing impacts the limits of detection.

As previously discussed (Limits of Detection by Read Mapping), the least abundant genomes to be detected by read mapping were vB_VpaS_sm032 and S-RSM4. Summed Illumina contigs from the pooled library mapped to 91 and 98 % of vB_VpaS_sm032 and S-RSM4, respectively. However, the longest individual contigs only covered a small fraction of the genomes (12 and 11 %, respectively). The average read depth for vB_VpaS_sm032 and S-RSM4 contigs was 10× over 98.7 % and 14× over 99.6 % of genome lengths, respectively, in the pooled Illumina library. Manual inspection of alignments revealed that breaks in the assemblies typically coincided with a drop in read coverage, which was often associated with a sudden and sharp change in mol%GC (either upwards or downwards; Fig. S3; Table S4) [60–62].

The longest genome to be recovered in a single contig, vB_Vpa_sm033 (320,253 bp), was assembled in Illumina-only, Illumina+PacBio and Illumina+ONT assemblies. The shortest genome to be recovered in a single contig, KUW1 (44,509 bp), was assembled in Illumina-only, Illumina+PacBio and Illumina+ONT assemblies. Whilst KUW1 was assembled from only one individual Illumina library, it was assembled in all the ONT+Illumina and PacBio+Illumina hybrid assemblies, suggests either type of long-read with short-reads can improve recovery of genomes.

### Assembly results – comparison of long-read assemblers

Prior to large-scale comparison of assemblers and read types, we examined the effect of polishing ONT assemblies, with long-reads using Medaka. After four rounds of polishing, assemblies produced using Unicycler, wtdbg2 and Canu observed decreases in SNP frequencies of 70, 30 and 1 %, respectively (Fig. S4A). Surprisingly, the Flye assemblies observed a 65 % increase in SNP frequencies (Fig. S4A). However, all ONT assemblies observed a decrease in INDELS. Unicycler, wtdbg2, Canu and Flye obtained 96, 90, 74 and 45 % decreases in the frequency of INDELs, respectively (Fig. S4B). Thus, we utilized medaka polished assemblies in further analyses.

It was possible to assemble a single genome that was representative of vB_Eco_mar001J1 and/or vB_Eco_mar002J2 from Illumina+ONT hybrid assemblies, rather than two genomes, perhaps unsurprisingly given there is >99 % ANI between them (Fig. S1). A single genome of vB_EcoS_swan01 was obtained from ONT-only assemblies produced using Canu, and Unicycler, as well as Illumina+ONT hybrid assemblies, which has ~80 % ANI with vB_Eco_SLUR29 (Fig. S1). However, the genome of vB_Eco_SLUR29 could not be resolved.

Long-read-only assemblies resulted in fewer complete genomes than short-reads alone (Fig. 3; Table S4). To identify the optimal long-read-only assemblies, we used the NGA50 statistic (Figs S5 & S6). While nine genomes were detected by mapping long-reads in at least one library, only eight are included in this analysis due to the very low coverage of vB_Eco_SLUR29 recovered from any assembly. For this comparison, we also included long-read assemblies that were polished with Illumina reads, as this was found to affect the results.

The NGA50 values averaged across the eight genomes and four libraries obtained from ONT assemblies were higher than those from PacBio. Again this varied depending on the assembler used. ONT reads assembled with Canu,Unicycler, Flye and wtdbg2 obtained average NGA50 values of 34, 28, 23 and 15 %, respectively, whereas PacBio reads obtained values of 16, 7 and 5 % for Canu, wtdbg2 and Flye assemblies, respectively. ONT reads assembled with Canu typically produced the longest alignments in relation to reference genomes. The performance of Canu with individual libraries was marginally higher than that in the pooled library, with the average NGA50 values as a proportion of genome length being 42, 33 and 31 % for individual libraries, and only 29 % for the pooled library (Figs S5 & S6). Conversely, the highest NGA50 values for ONT reads assembled with wtdbg2 were obtained from the pooled library (19 %), and 13, 14 and 16 % from individual libraries (Figs S5 & S6). Thus, pooling reads was both detrimental or advantageous, depending on the assembler used.

To determine if the long-read assemblies were failing due to high-sequencing depth, reads mapping to genomes with ≥100×coverage were extracted, randomly downsampled to different coverage levels, and re-assembled. For both ONT and PacBio, and all assemblers used, downsampling the reads prior to re-assembly led to more complete assemblies (Fig. 4; Table S5). Furthermore, successful assemblies using PacBio reads with Unicycler were only obtained after downsampling.

### Assembly results – SNPs, INDELs and mis-assemblies

To investigate the fidelity of assemblies, we compared assembled contigs to the mock community reference genomes to identify the frequency of SNPs and INDELs per 100 kb. Assemblies with the highest fidelity would be expected to have the smallest number of SNPs and INDELS compared to the known references. Both SNPs and INDELs were calculated for genomes where ≥50 % of the genome was covered by contigs. When using one technology only, Illumina reads resulted in the lowest number of SNPs per 100 kb (468) with ONT long-read-only assemblies having the highest number of SNPs (662–853 per assembler). The number of SNPs in long-read assemblies was also dependent on the assembler used. Using ONT reads with Unicycler (662) resulted in fewer SNPs than wtdbg2 (780), Canu (840) or Flye (853) (Figs S7A & S8). A different pattern was observed for PacBio reads, with Canu performing best (522), followed by Flye (901) and wtdbg2 (974) (Figs S7A & S8).

A similar pattern of results was observed for the number of INDELs per 100 kb, although a much larger difference between the different assemblers was observed. PacBio assemblies produced using wtdbg2 had the highest number of INDELs (519), although this was far lower when using Flye (144) and Canu (25). Similarly, ONT assemblies produced using wtdbg2 had a far higher number of INDELs (130) than those produced using Unicycler (114), Flye (88) and Canu (37). Illumina-only assemblies had the smallest number of INDELs (15) (Figs S7B & S9).

### Effect of short-read polishing on long-read assemblies on SNPs, INDELs and orf prediction

**Fig. 5.**
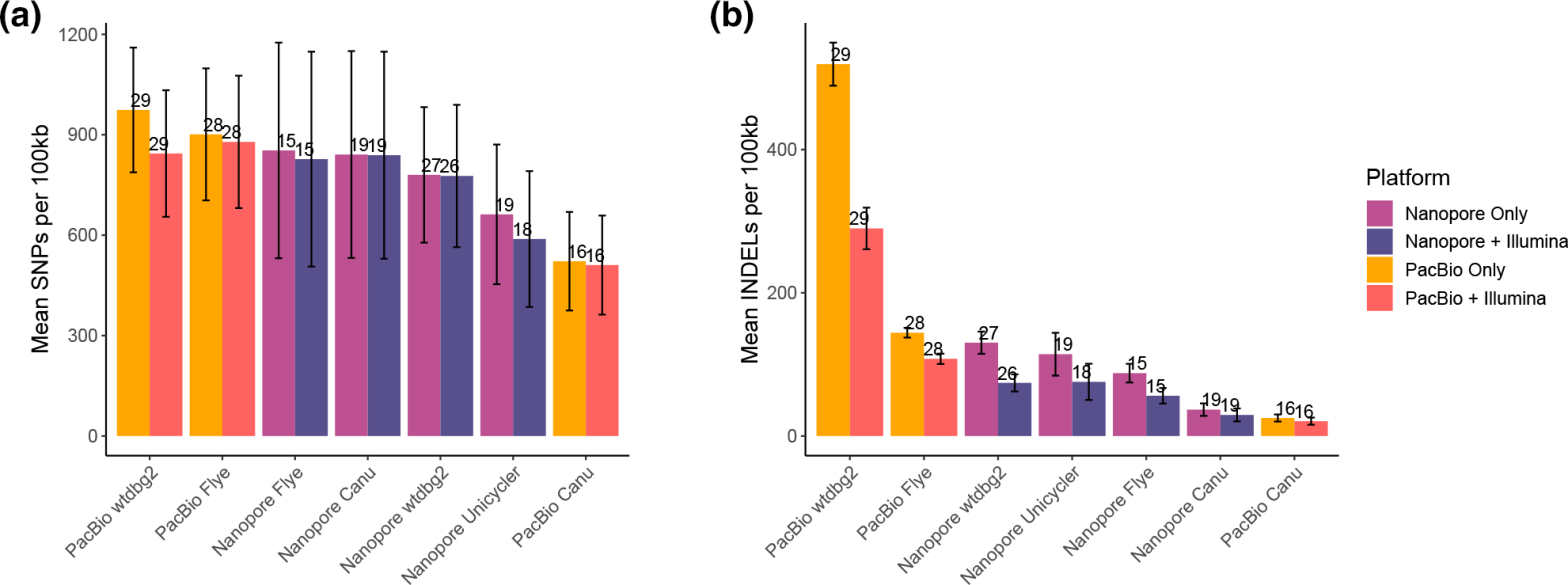
Effect of polishing on error rate. SNPs and INDELs were identified in contigs from nanopore only, nanopore contigs polished with illumina reads, PacBio only and PacBio contigs polished with illumina reads. The number of SNPs (**a**) and INDELSs (**b**) are expressed as the number per 100 kb of reference genome, where at least 50 % of the reference genome was recovered by contigs, where at least 50 % of the reference genome was recovered by contigs. Error bars are standard error of the mean, and the number above the bar indicates the number of genomes included in the mean calculation [from a total possible maximum of 60 (15 genomes, 4 assemblies)].

Using short-reads to polish contigs produced from long-read assemblies generally reduced the number of SNPs per 100 kb, although this was dependent on the specific assembly. Polishing ONT assemblies produced with Unicycler, Flye, wtdbg2 and Canu decreased the frequency of SNPs by 11, 3, 0.4 and 0.2 %, respectively (Fig. 5a). All PacBio assemblies observed decreases in SNP frequencies after polishing with Illumina reads, with reductions of 13, 2 and 2 % for wtdbg2, Flye and Canu, respectively (Fig. 5a). The effect of polishing on the frequency of INDELs was more apparent, with all assemblies obtaining a decrease to the number of INDELs. The PacBio assemblies had both the highest and lowest number of INDELs prior to polishing (Fig. 5b), which were reduced by 44, 25 and 18 % for wtdbg2, Flye and Canu, respectively, following polishing with Illumina reads. Large decreases were also observed for ONT assemblies, with wtdbg2, Flye, Unicycler and Canu assembly INDEL frequencies being reduced by 43, 36, 34 and 20 %, respectively (Fig. 5b).

**Fig. 6.**
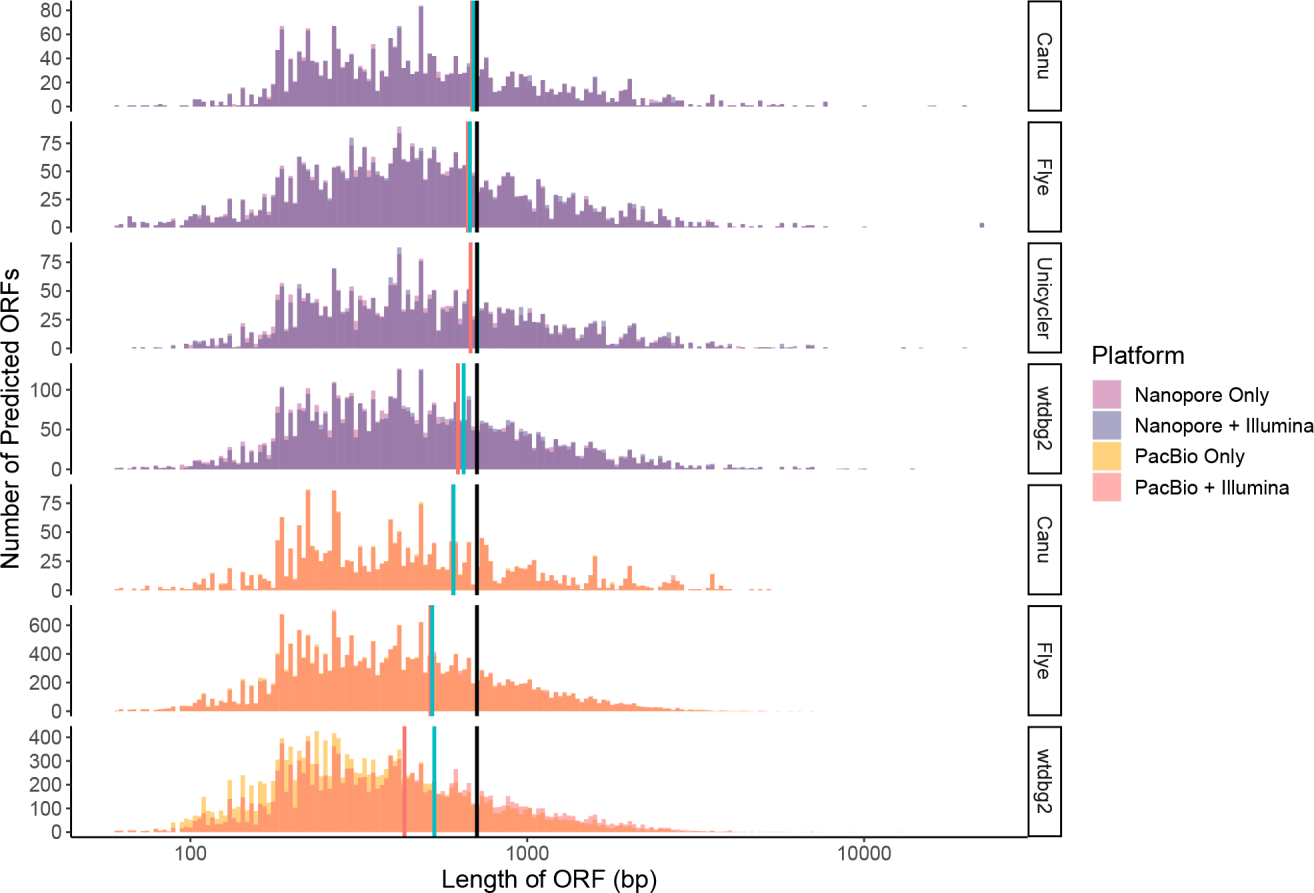
The effect of polishing long-read assemblies on predicted ORF lengths. ORF length was calculated from Nanopore-only contigs, Nanopore contigs polished with Illumina reads, PacBio-only contigs and PacBio contigs polished with Illumina reads. With contigs obtained using Flye, wtdbg2 and Unicycler assemblers. Histograms show the distribution of predicted ORF length for sequencing type (Nanopore or PacBio) and assembly algorithm. The expected mean ORF length from the reference genomes is represented as a solid vertical line (709 bp; black), compared to the mean value before (red) and after (blue) polishing.

As assembly errors can have an effect on ORF prediction and functional annotation [63], we investigated the number and length of predicted ORFs on contigs, which mapped to reference genomes before and after polishing. Polishing with short-reads increased the mean ORF length for all assemblies, although this varied with assembler used. PacBio assemblies observed both the largest and smallest increases to mean ORF length following polishing with Illumina reads. PacBio assemblies produced using wtdbg2 saw a 22.7 % increase in mean ORF length, versus 1.3 and 0.3 % for Flye and Canu, respectively (Fig. 6). ONT assemblies had increases of 4.8, 4.0 1.4 and 1.0 % in mean ORF length for Unicycler, wtdbg2, Flye and Canu, respectively (Fig. 6). All combinations of reads and assemblers were smaller than expected values calculated from the 15 reference genomes. With Canu, Unicycler and Flye assemblies of Nanopore, polished with Illumina closest to the expected mean ORF length (Fig. 6).

Overall, long-read-only assemblies had a high frequency of SNPs and INDELs, hybrid assemblies produced with Unicycler that combined Illumina reads with ONT or PacBio reads obtained SNP and INDEL levels comparable to Illumina-only assemblies (Fig. S7B). Thus, there is a clear advantage to using a hybrid assembly as it reduces the number of errors.

### Effect of polishing long-read assemblies on viral prediction

Many viral prediction programmes use similarity of predicted proteins to known hallmark proteins for virus prediction. Thus, truncated proteins may alter the ability to predict viral contigs from viromes. To test if truncated proteins affect virus prediction, we compared vibrant [52], which in part uses predicted proteins, and DeepVirFinder [53] a K-mer based prediction system on all assembled contigs. Although we utilized purified phage isolates to create the mock community, up to 20 % of the reads from Illumina libraries did not map to the reference phage genomes. With a 19.4+/–1.5 % of reads mapping to host bacterial genomes (Table S7) Therefore, we utilized this unfortunate level of contaminating host bacterial DNA for benchmarking viral prediction. To determine how many predictions represented ‘true’ viral predictions, we mapped the predicted vOTUs against the reference genomes. Despite differences in mean gene length before and after polishing long-read-only assemblies with Illumina reads, there was no difference between the number of predicted vOTUs with vibrant. There was also no difference observed for the number of vOTUs predicted with DVF. Reassuringly, the majority (~99 %) of predicted vOTUs mapped to the input genomes and were deemed to be true predictions (Fig. S10; Table S8).

### Effect of sequencing technology on predicted virome diversity

**Fig. 7.**
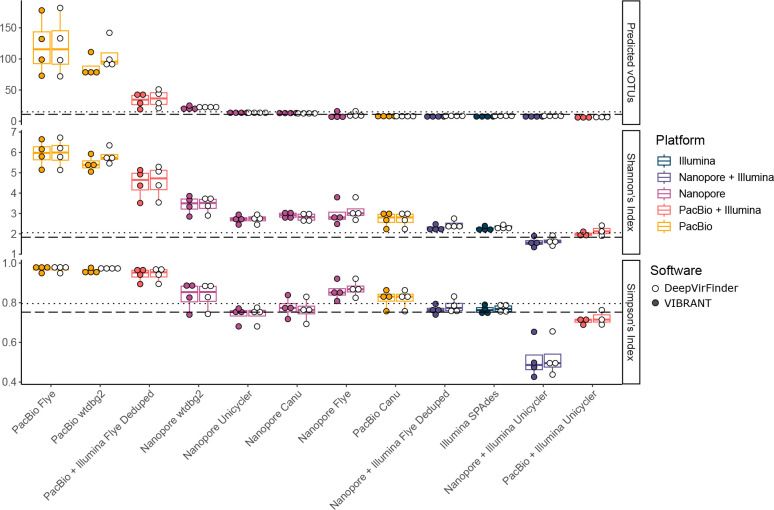
The effect of sequencing platform and assembler on diversity estimates. Boxplots showing the number of predicted vOTUs for mock virome analysis (top), and Shannon’s index (middle) and Simpson’s index (bottom) alpha diversity measures. Separate boxes are included for viral predictions based upon *k*-mer frequencies (DeepVirFinder; colour filled dots) and predicted proteins (vibrant; white dots). Dotted lines indicate true values for mock virome input, and dashed lines indicate true values excluding genomes that were not detected by read mapping in any library.

Having established there was little difference between the two viral prediction pipelines on our datasets, we utilized the output of DeepVirFinder and vibrant predictions to assess how diversity statistics of the mock community varied with sequencing technology and assembly. Given the diversity of phages and the lack of core-genes found in all phages, there is always a balance between using all contigs found within a sample and only complete phage genomes, to define a viral population (vOTU). The use of all non-redundant contigs is known to overestimate viral diversity [64] and using only complete genomes provide an underestimate. Thus, we utilized the now standard threshold of Roux *et al*., where a phage population is considered as vOTU for contigs ≥10 kb, that are detected by read mapping across ≥70 % of its length [55]. Alpha diversity was assessed using the predicted number of vOTUs, Shannons’s index and Simpson’s index; overall the same general trend was observed for each metric (Fig. 7; Table S9).

When using long-read assemblies there was an overprediction of alpha diversity (ONT: median vOTUs 13.5, PacBio: median vOTUs 79; expected vOTUs 11). In contrast, when using Illumina, and Illumina+ONT/PacBio direct hybrid assemblies there was an underestimation of alpha diversity (ONT-Illumina hybrid: median vOTUs 8, PacBio-Illumina hybrid: median vOTUs 6.5, Illumina: median vOTUs 7). Within this general trend, there was further variation with the method used for assembly. While the use of Illumina or Illumina+ONT/PacBio reads generally led to an underestimation of diversity, it was closer to the known diversity measurement than the use of long-reads alone. When using long-reads alone, using ONT reads assembled with Unicycler gave the closest estimate of the true diversity of the mock community.

## Discussion

The use of long-read sequencing technologies is becoming increasingly common for the sequencing of metagenomics samples, in particular those that focus on the bacterial community. A number of studies have demonstrated the advantage of long-reads in assembling complete genomes from a variety of samples [26, 65–67]. There have also been a number of studies benchmarking the assembly and/or recovery of bacteria from mock communities using long-reads [68, 69], along with benchmarking of assembly algorithms for prokaryotic genomes (excluding phages) [70, 71]. However, there are no such comprehensive studies that have directly compared Illumina, ONT, and PacBio sequencing technologies for the study of viromes.

Using a mapping approach, only 11 of the 15 genomes could be detected in the deepest sequenced samples (Illumina). Phage S-RSM4, was the least abundant phage to be detected by a mapping approach. Thus, the absence of phage CDHM1, which was ~10× less abundant than phage S-RSM4 is likely as a result of sequencing depth being limiting in all technologies. PhiX-174, a ssDNA, phage was not detected with any technology. Non-detection with Illumina is consistent with the use of a NexteraXT library preparation, which requires dsDNA for transposon insertion (the PhiX Illumina control has adapters ligated). The lack of detection with long-read technologies could be ascribed to lower sequencing depth with these approaches. Why the more abundant phages HP1 and vB_Eco_mar005P1 could not be detected is unclear. Given the unexpectedly high levels of host DNA in the pool, it is possible these phages had higher levels of host DNA and the absolute level of phage DNA added was less than calculated.

Previous benchmarking of short-read assemblers has demonstrated minimal differences in genome recovery of phage genomes when comparing multiple assemblers on a mock viral community [55]. For this reason, we chose only one short-read assembly algorithm: SPAdes. For long-read assembly, we chose four frequently used approaches of Unicycler (miniasm+racon), Flye, Canu and wtdgb2, as well as using Unicycler for a direct hybrid assembly. For long-read sequencing alone, we were unable to obtain assemblies from PacBio reads with Unicycler, even when combining all three libraries, suggesting it was not a lack of coverage.

When using a single sequencing technology, Illumina performed best in the recovery of completely assembled phage genomes. Utilizing a hybrid approach increased the number of genomes that could be assembled, with ONT+Illumina reads assembled with Unicycler (miniasm+racon) recovering the largest number of genomes, whereas the addition of PacBio reads did not result in the same increased recovery of genomes. However, this may well be due to the reduced yield of PacBio reads compared to ONT reads. Thus, increased yield of PacBio data might improve this metric. However, it was clear for both ONT and PacBio that very high coverage of specific genomes within a sample was detrimental to genome assembly, with subsampling of reads increasing the length of the contig recovered. Thus, the use of specific viral binning software such as vRhyme [72] and mapping of reads to these bins and subsequent down sampling of reads may improve genome assembly. Alternatively the use of digital normalization of reads may improve recovery of longer contigs, as observed in other systems [73]. For phages, generally 50–100×coverage produced the longest contigs.

The combination of long- and short-reads improving recovery of assembled genomes is consistent with previous benchmarking of a mock viral community using a virION (Viral, long-read metagenomics via MinION sequencing) approach [15]. Unlike the virION approach, we were only able to assemble a single genome with ONT read-only assemblies. However, direct comparison between the studies is difficult given the different phages used in each mock community. Here, we utilized MDA application to provide sufficient material for long-read sequencing, whereas the virION utilizes PCR to provide sufficient material [15, 24]. The virION approach has comprehensively demonstrated that the relative abundance of phages is maintained due to the LASL-PCR approach [15, 24]. Here, we observed a strong correlation in the abundance of phages in the un-amplified Illumina viromes and amplified long-read viromes. However, we are cautious in the interpretation of this data. The DNA from a ssDNA phage (PhiX174) was spiked into our mock community at a deliberately low level, as we wanted to avoid flooding our amplified DNA with ssDNA given known biases of MDA. However, given the lack of detection of PhiX174 in any long-read samples, we may have been overly cautious in the amount added. Thus, when ssDNA phages are present in a community, it is likely the biases observed previously are still likely to hold true [20–22].

When assessing any individual sequencing technology alone, the lowest number of SNPs or INDELs observed when using Illumina reads, with ONT assemblies having a larger number of SNPs, and in particular INDELs, compared to PacBio assemblies. Thus, the short-read assembly produced the highest fidelity genomes. Both INDELs and SNPs were also affected by the method used for assembly. For ONT reads, Flye produced assemblies with the lowest number of INDELs or SNPs compared to wtdgb2 and Unicycler (miniasm+racon). It is likely for ONT data that the number of SNPs and INDELs will further decrease with improvements in accuracy reported for both R10 flow cells and the latest base-calling algorithms that have been developed since this data was collected and has been observed for bacterial genomes [74, 75], as this data was generated with R9 flow cells. In contrast, Flye assemblies of PacBio reads had the lowest number of SNPs, but the highest number of INDELs. With the release of HiFi reads from PacBio [76], the number of SNP and INDELS is also likely to decrease in the future. Thus, the choice of assembly method should be adjusted for the type of long-reads being used. The addition of short-reads to polish the long-read assemblies resulted in a reduction of both SNPs and INDELs, as has been observed in other studies [15, 23, 27].

Whilst the combination of both short Illumina reads with long-reads resulted in the ‘best’ overall assemblies, it may well not be feasible to sequence samples with both technologies. Therefore, we treated the assemblies from multiple approaches to assess how the different approaches affected the predicted diversity of the sample. Although polishing long-read assemblies had a significant impact on reducing the number of SNPs and INDELs, there was minimal effect on the number of predicted contigs that were viral when using DeepVirFinder for prediction. Thus, the choice of sequencing technology may have ramifications for downstream choices in viral prediction software.

### Conclusions

We have benchmarked Illumina, ONT, and PacBio sequencing platforms for virome analysis using a number of read and assembler combinations and offer recommendations for the community: (i) if only using one sequencing platform, Illumina performs best at genome recovery and has the lowest error rates; (ii) the addition of long-reads to Illumina reads improves the assembly of lowly abundant genomes, particularly ONT; (iii) whilst long-read assemblies, particularly ONT, have higher error frequencies, polishing with Illumina reads can reduce these errors to levels comparable with Illumina-only assemblies; (iv) down-sampling of long-reads may aid assembly; and (v) the choice of sequencing platform should be considered when making downstream analysis decisions, such as assembler algorithm and viral prediction software.

## Supplementary Data

Supplementary material 1

Supplementary material 2
