# Supplementary material for: The long and short of it: benchmarking viromics using Illumina, Nanopore and PacBio sequencing technologies
Source: Microb Genom. 2024 Feb 20;10(2):001198. doi: 10.1099/mgen.0.001198 (PMC10926689; doi:10.1099/mgen.0.001198)
Supplement: Supplementary material 1 [file mgen-10-1198-s001.pdf]

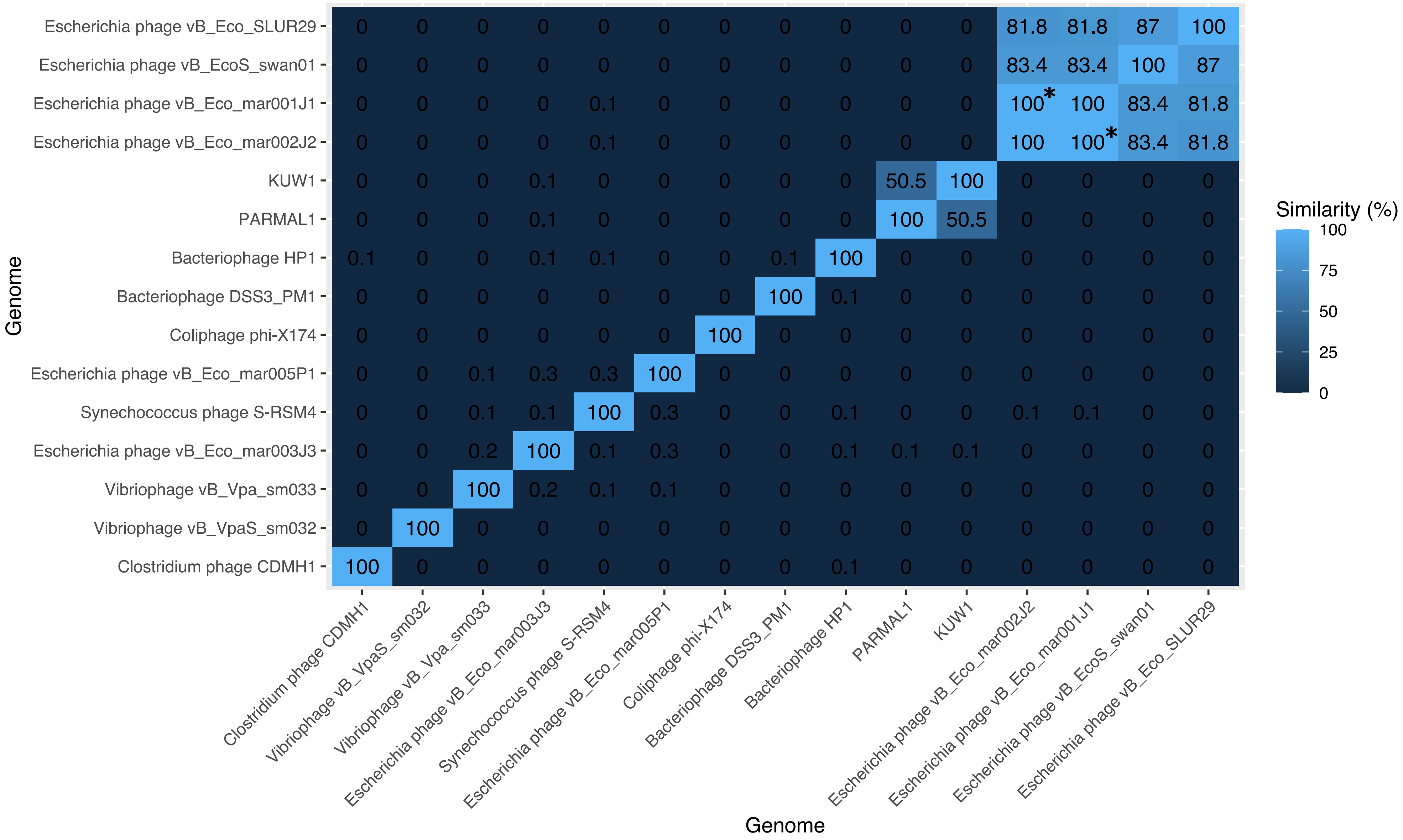

Escherichia phage vB_Eco_SLUR29
0 0 0 0 0 0 0 0 0 0 0 81.8 81.8 87 100
Escherichia phage vB_EcoS_swan01
0 0 0 0 0 0 0 0 0 0 0 83.4 83.4 100 87
Escherichia phage vB_Eco_mar001J1
0 0 0 0 0.1 0 0 0 0 0 0 100* 100 83.4 81.8
Escherichia phage vB_Eco_mar002J2
0 0 0 0 0.1 0 0 0 0 0 0 100 100* 83.4 81.8
KUW1
0 0 0 0.1 0 0 0 0 0 50.5 100 0 0 0 0
PARMAL1
0 0 0 0.1 0 0 0 0 0 100 50.5 0 0 0 0
Bacteriophage HP1
0.1 0 0 0.1 0.1 0 0 0.1 100 0 0 0 0 0 0
Bacteriophage DSS3_PM1
0 0 0 0 0 0 0 100 0.1 0 0 0 0 0 0
Coliphage phi-X174
0 0 0 0 0 0 100 0 0 0 0 0 0 0 0
Escherichia phage vB_Eco_mar005P1
0 0 0.1 0.3 0.3 100 0 0 0 0 0 0 0 0 0
Synechococcus phage S-RSM4
0 0 0.1 0.1 100 0.3 0 0 0.1 0 0 0.1 0.1 0 0
Escherichia phage vB_Eco_mar003J3
0 0 0.2 100 0.1 0.3 0 0 0.1 0.1 0.1 0 0 0 0
Vibriophage vB_Vpa_sm033
0 0 100 0.2 0.1 0.1 0 0 0 0 0 0 0 0 0
Vibriophage vB_VpaS_sm032
0 100 0 0 0 0 0 0 0 0 0 0 0 0 0
Clostridium phage CDMH1
100 0 0 0 0 0 0 0 0.1 0 0 0 0 0 0
Genome
Clostridium phage CDMH1
Vibriophage vB_VpaS_sm032
Vibriophage vB_Vpa_sm033
Escherichia phage vB_Eco_mar003J3
Synechococcus phage S-RSM4
Escherichia phage vB_Eco_mar005P1
Coliphage phi-X174
Bacteriophage DSS3_PM1
Bacteriophage HP1
PARMAL1
KUW1
Escherichia phage vB_Eco_mar002J2
Escherichia phage vB_Eco_mar001J1
Escherichia phage vB_EcoS_swan01
Escherichia phage vB_Eco_SLUR29
Genome
Similarity (%)
100
75
50
25
0

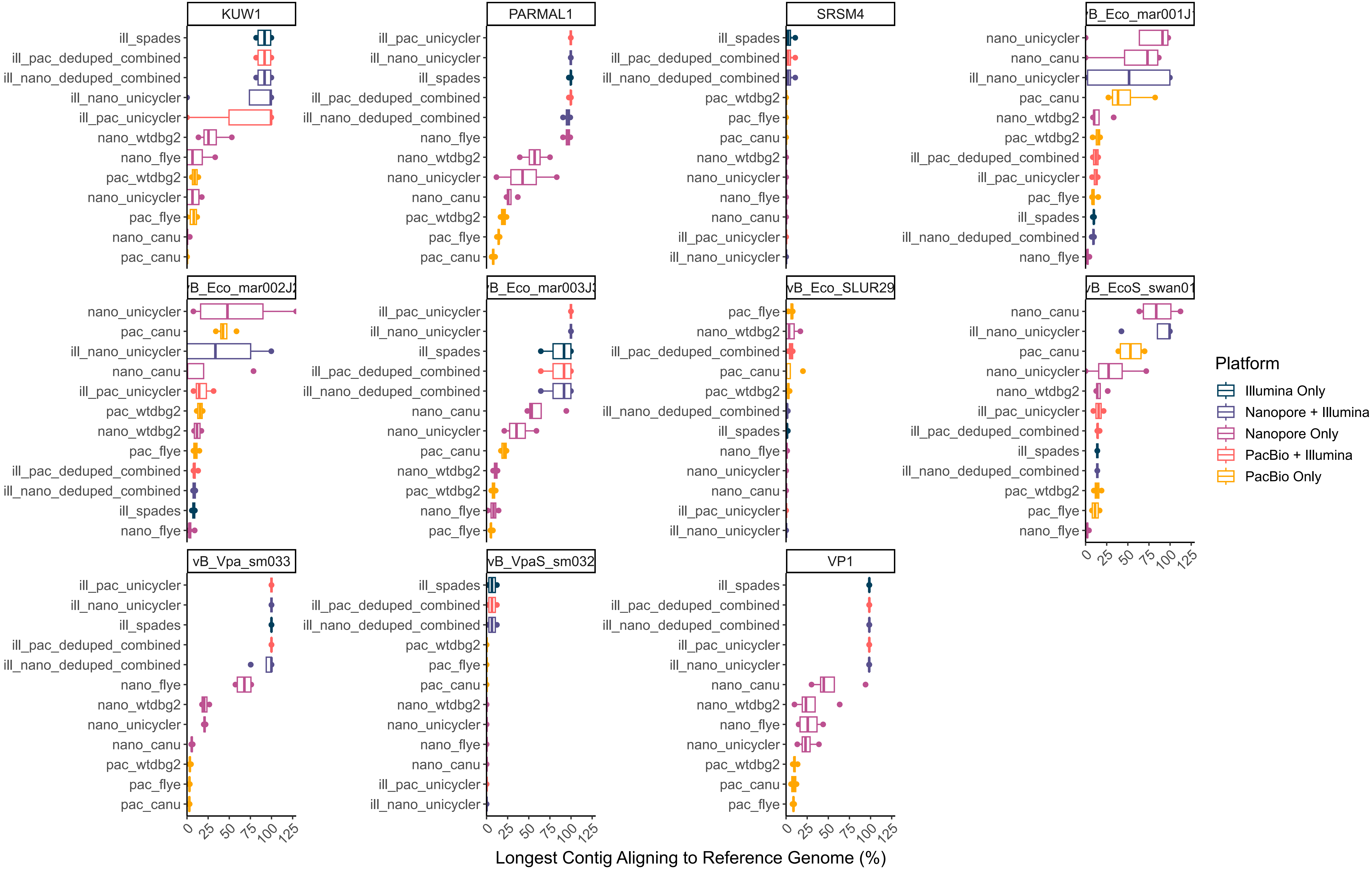

KUW1
PARMAL1
SRSM4
vB_Eco_mar001J1
vB_Eco_mar002J2
vB_Eco_mar003J3
vB_Eco_SLUR29
vB_EcoS_swan01
vB_Vpa_sm033
vB_VpaS_sm032
VP1
ill_spades
ill_pac_deduped_combined
ill_nano_deduped_combined
ill_nano_unicycler
ill_pac_unicycler
nano_wtdbg2
nano_flye
pac_wtdbg2
nano_unicycler
pac_flye
nano_canu
pac_canu
0
25
50
75
100
125
Platform
Illumina Only
Nanopore + Illumina
Nanopore Only
PacBio + Illumina
PacBio Only
Longest Contig Aligning to Reference Genome (%)

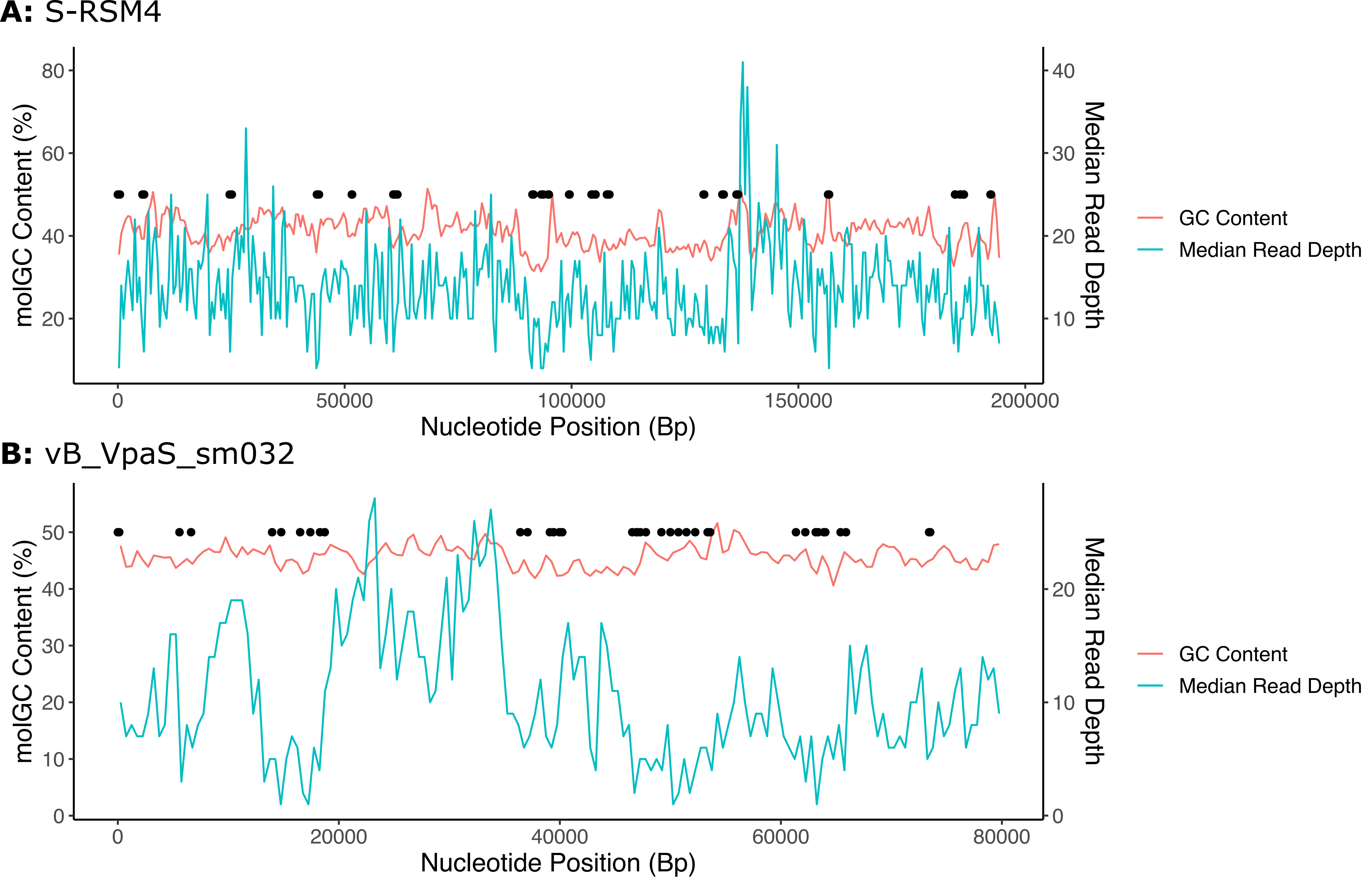
A: S-RSM4
molGC Content (%)
Median Read Depth
Nucleotide Position (Bp)
GC Content
Median Read Depth
0
50000
100000
150000
200000
20
40
60
80
10
20
30
40
B: vB_VpaS_sm032
molGC Content (%)
Median Read Depth
Nucleotide Position (Bp)
GC Content
Median Read Depth
0
20000
40000
60000
80000
0
10
20
30
40
50
0
10
20

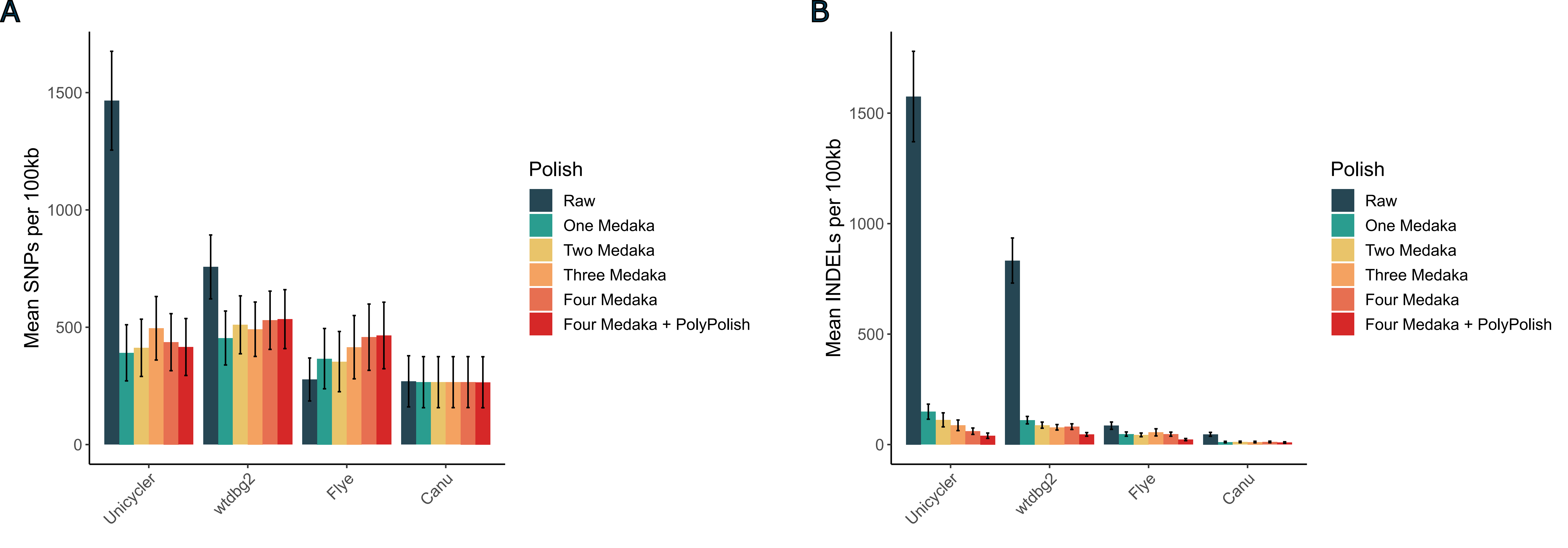
A
Mean SNPs per 100kb
0
500
1000
1500
Unicycler
wtdbg2
Flye
Canu
Polish
Raw
One Medaka
Two Medaka
Three Medaka
Four Medaka
Four Medaka + PolyPolish
B
Mean INDELs per 100kb
0
500
1000
1500
Unicycler
wtdbg2
Flye
Canu
Polish
Raw
One Medaka
Two Medaka
Three Medaka
Four Medaka
Four Medaka + PolyPolish

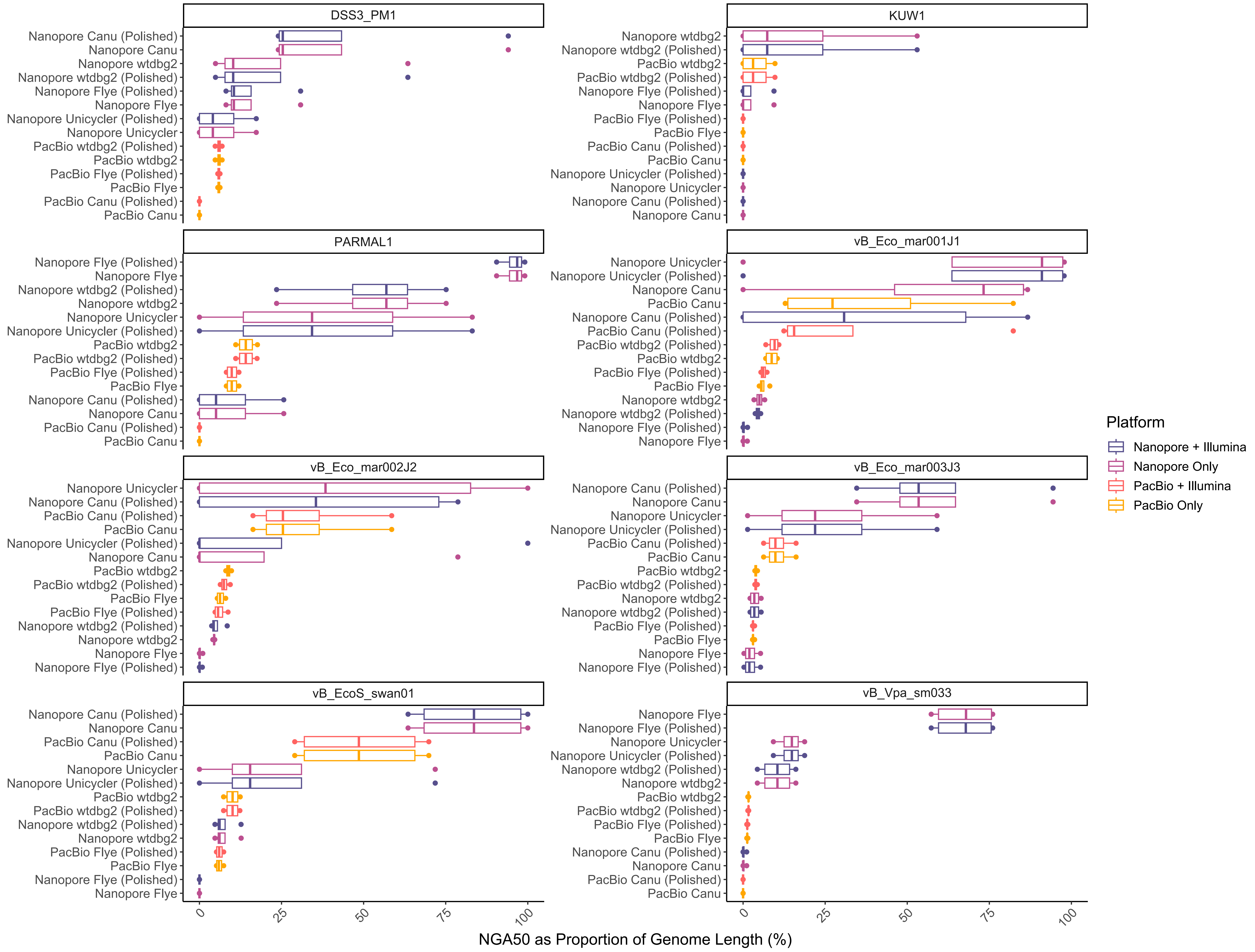
DSS3_PM1
Nanopore Canu (Polished)
Nanopore Canu
Nanopore wtdbg2
Nanopore wtdbg2 (Polished)
Nanopore Flye (Polished)
Nanopore Flye
Nanopore Unicycler (Polished)
Nanopore Unicycler
PacBio wtdbg2 (Polished)
PacBio wtdbg2
PacBio Flye (Polished)
PacBio Flye
PacBio Canu (Polished)
PacBio Canu
KUW1
Nanopore wtdbg2
Nanopore wtdbg2 (Polished)
PacBio wtdbg2
PacBio wtdbg2 (Polished)
Nanopore Flye (Polished)
Nanopore Flye
PacBio Flye (Polished)
PacBio Flye
PacBio Canu (Polished)
PacBio Canu
Nanopore Unicycler (Polished)
Nanopore Unicycler
Nanopore Canu (Polished)
Nanopore Canu
PARMAL1
Nanopore Flye (Polished)
Nanopore Flye
Nanopore wtdbg2 (Polished)
Nanopore wtdbg2
Nanopore Unicycler
Nanopore Unicycler (Polished)
PacBio wtdbg2
PacBio wtdbg2 (Polished)
PacBio Flye (Polished)
PacBio Flye
Nanopore Canu (Polished)
Nanopore Canu
PacBio Canu (Polished)
PacBio Canu
vB_Eco_mar001J1
Nanopore Unicycler
Nanopore Unicycler (Polished)
Nanopore Canu
PacBio Canu
Nanopore Canu (Polished)
PacBio Canu (Polished)
PacBio wtdbg2 (Polished)
PacBio wtdbg2
PacBio Flye (Polished)
PacBio Flye
Nanopore wtdbg2
Nanopore wtdbg2 (Polished)
Nanopore Flye (Polished)
Nanopore Flye
vB_Eco_mar002J2
Nanopore Unicycler
Nanopore Canu (Polished)
PacBio Canu (Polished)
PacBio Canu
Nanopore Unicycler (Polished)
Nanopore Canu
PacBio wtdbg2
PacBio wtdbg2 (Polished)
PacBio Flye
PacBio Flye (Polished)
Nanopore wtdbg2 (Polished)
Nanopore wtdbg2
Nanopore Flye
Nanopore Flye (Polished)
vB_Eco_mar003J3
Nanopore Canu (Polished)
Nanopore Canu
Nanopore Unicycler
Nanopore Unicycler (Polished)
PacBio Canu (Polished)
PacBio Canu
PacBio wtdbg2
PacBio wtdbg2 (Polished)
Nanopore wtdbg2
Nanopore wtdbg2 (Polished)
PacBio Flye (Polished)
PacBio Flye
Nanopore Flye
Nanopore Flye (Polished)
vB_EcoS_swan01
Nanopore Canu (Polished)
Nanopore Canu
PacBio Canu (Polished)
PacBio Canu
Nanopore Unicycler
Nanopore Unicycler (Polished)
PacBio wtdbg2
PacBio wtdbg2 (Polished)
Nanopore wtdbg2 (Polished)
Nanopore wtdbg2
PacBio Flye (Polished)
PacBio Flye
Nanopore Flye (Polished)
Nanopore Flye
vB_Vpa_sm033
Nanopore Flye
Nanopore Flye (Polished)
Nanopore Unicycler
Nanopore Unicycler (Polished)
Nanopore wtdbg2 (Polished)
Nanopore wtdbg2
PacBio wtdbg2
PacBio wtdbg2 (Polished)
PacBio Flye (Polished)
PacBio Flye
Nanopore Canu (Polished)
Nanopore Canu
PacBio Canu (Polished)
PacBio Canu
0
25
50
75
100
NGA50 as Proportion of Genome Length (%)
Platform
Nanopore + Illumina
Nanopore Only
PacBio + Illumina
PacBio Only

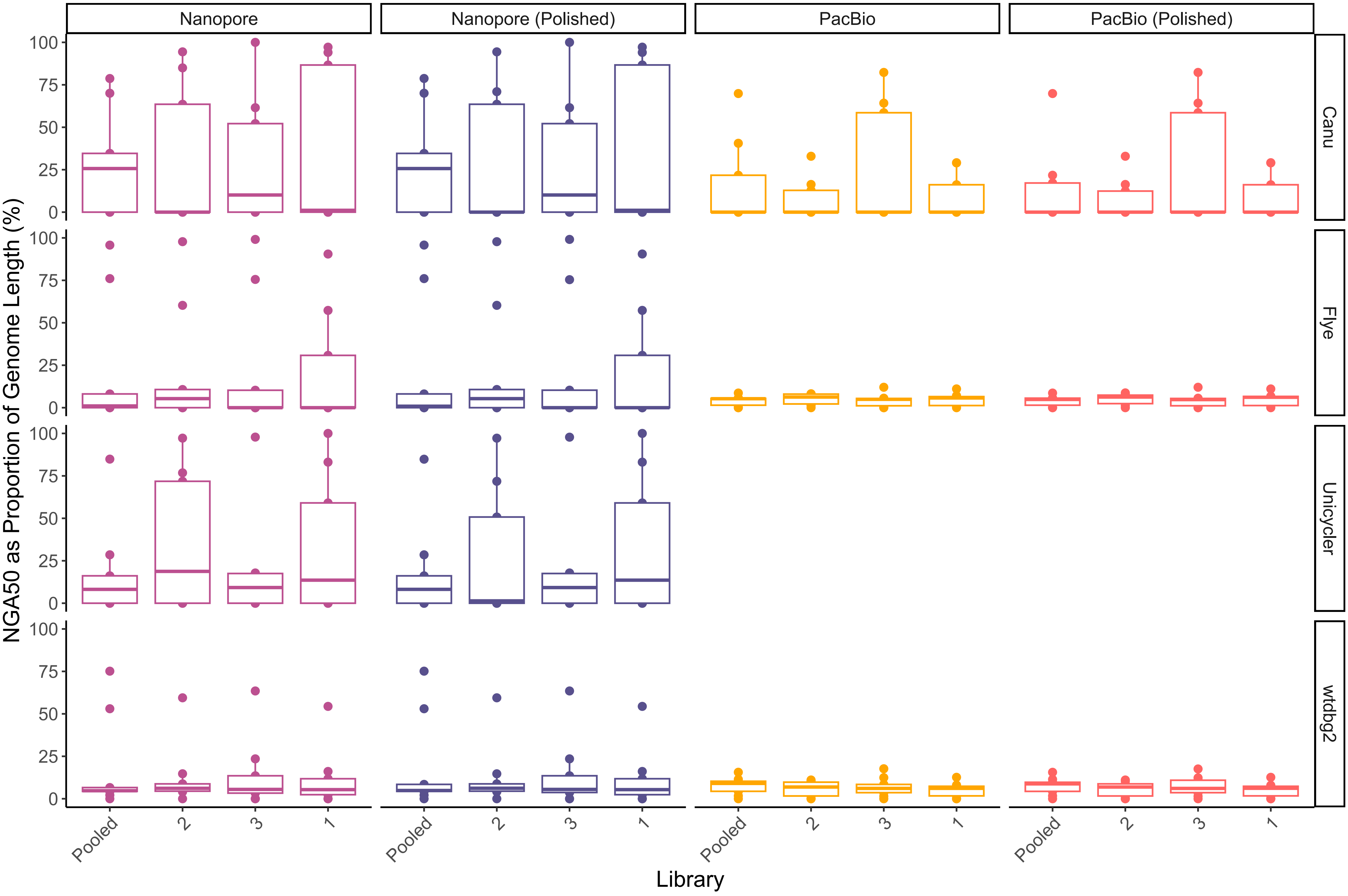

Nanopore
Nanopore (Polished)
PacBio
PacBio (Polished)
Canu
Flye
Unicycler
wtdbg2
NGA50 as Proportion of Genome Length (%)
0
25
50
75
100
Pooled
2
3
1
Library

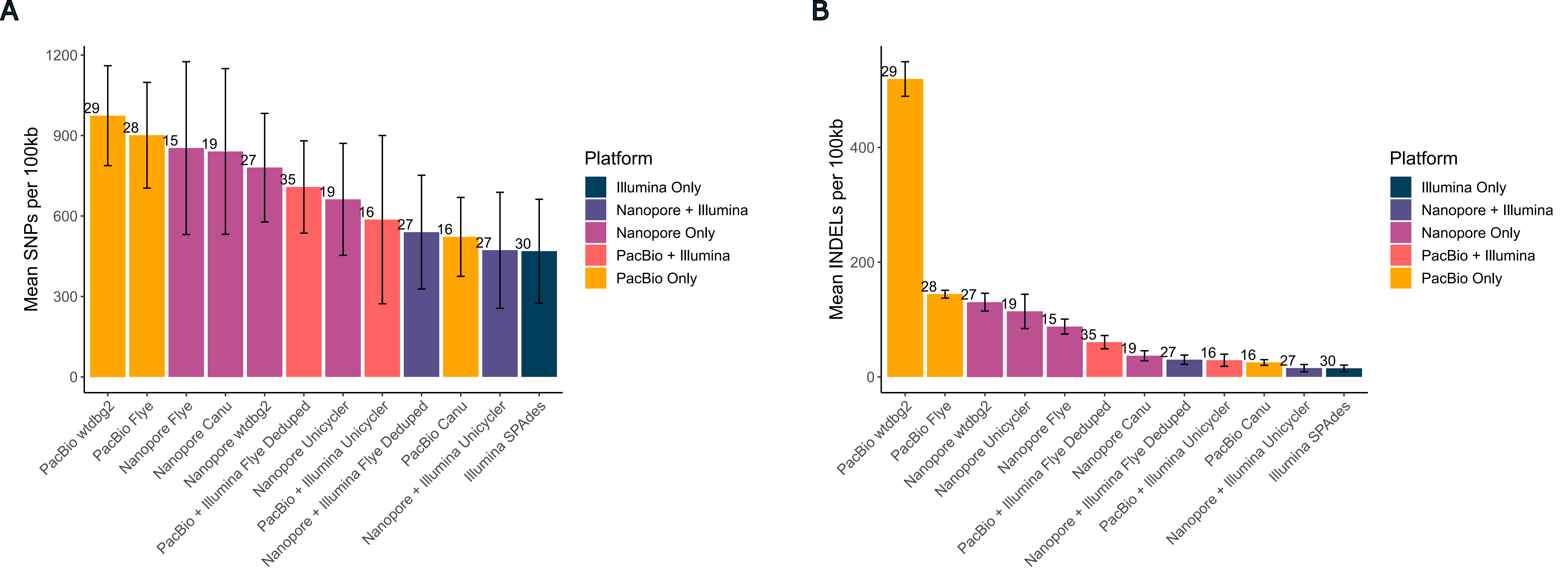
A
B
Mean SNPs per 100kb
Mean INDELs per 100kb
Platform
Illumina Only
Nanopore + Illumina
Nanopore Only
PacBio + Illumina
PacBio Only
PacBio wtdbg2
PacBio Flye
Nanopore Flye
Nanopore Canu
Nanopore wtdbg2
PacBio + Illumina Flye Deduped
Nanopore Unicycler
PacBio + Illumina Unicycler
Nanopore + Illumina Flye Deduped
PacBio Canu
Nanopore + Illumina Unicycler
Illumina SPAdes

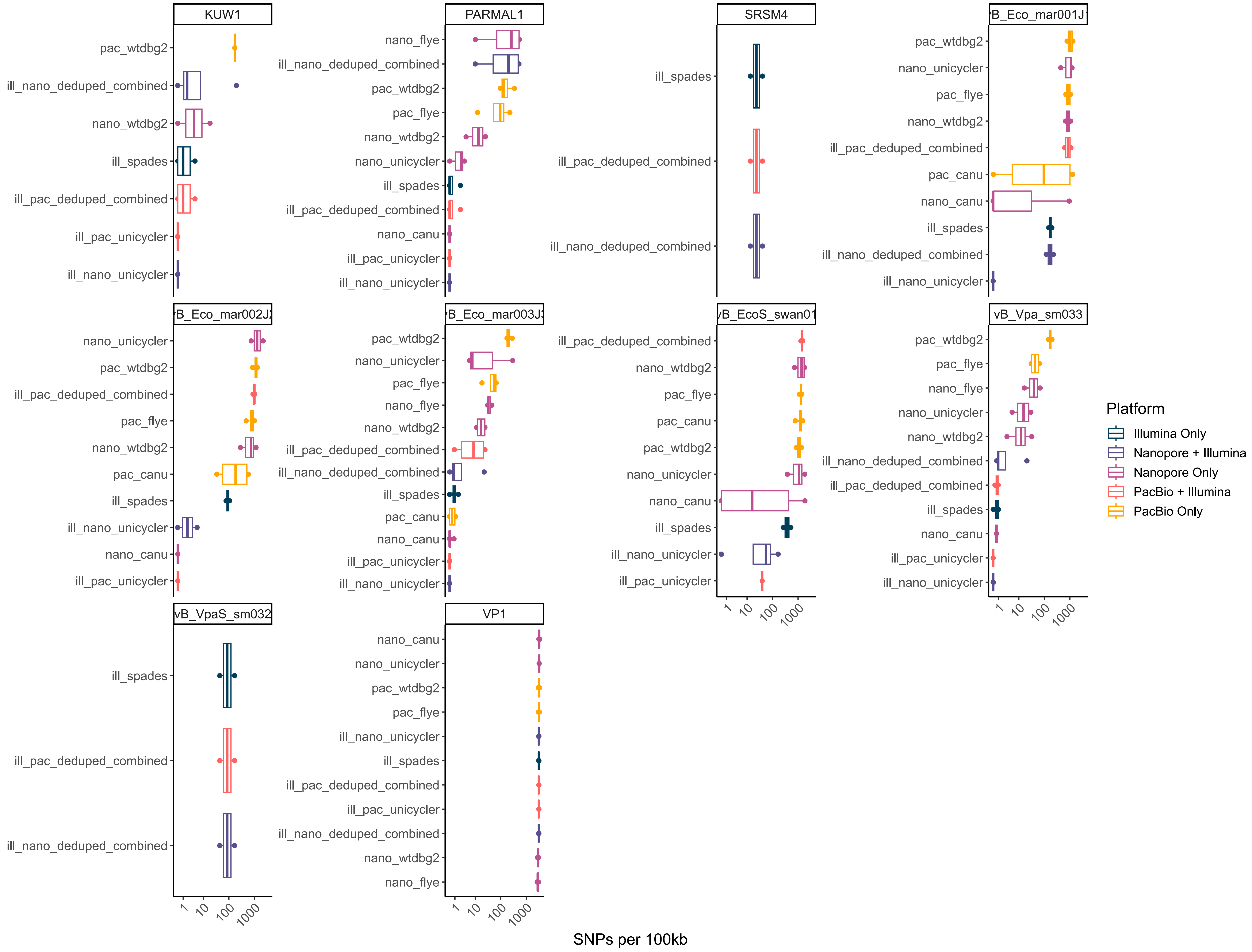

KUW1
PARMAL1
SRSM4
vB_Eco_mar001J1
vB_Eco_mar002J2
vB_Eco_mar003J3
vB_EcoS_swan01
vB_Vpa_sm033
vB_VpaS_sm032
VP1
pac_wtdbg2
ill_nano_deduped_combined
nano_wtdbg2
ill_spades
ill_pac_deduped_combined
ill_pac_unicycler
ill_nano_unicycler
nano_flye
pac_flye
nano_unicycler
nano_canu
pac_canu
1
10
100
1000
SNPs per 100kb
Platform
Illumina Only
Nanopore + Illumina
Nanopore Only
PacBio + Illumina
PacBio Only

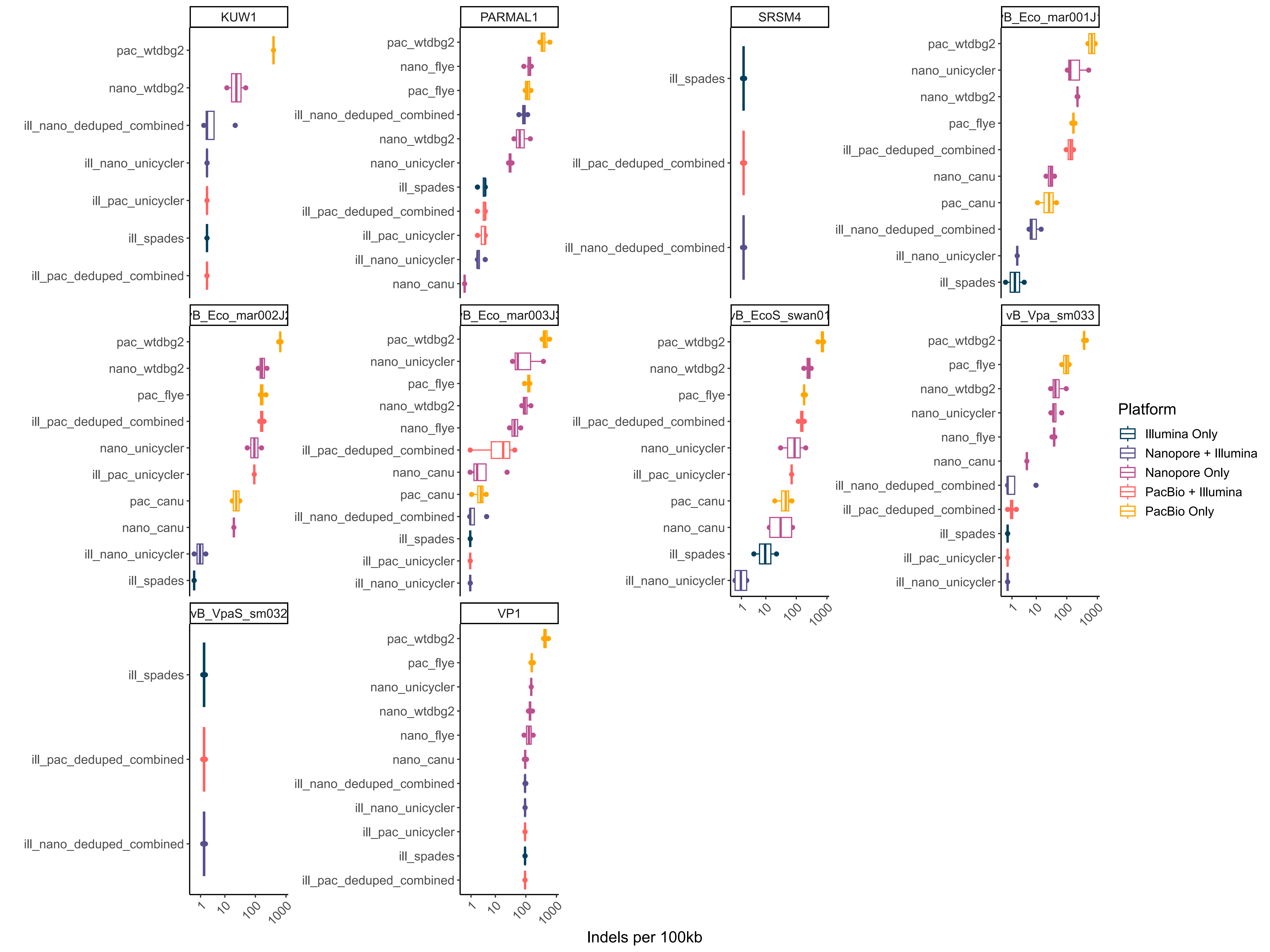
KUW1
pac_wtdbg2
nano_wtdbg2
ill_nano_deduped_combined
ill_nano_unicycler
ill_pac_unicycler
ill_spades
ill_pac_deduped_combined
PARMAL1
pac_wtdbg2
nano_flye
pac_flye
ill_nano_deduped_combined
nano_wtdbg2
nano_unicycler
ill_spades
ill_pac_deduped_combined
ill_pac_unicycler
ill_nano_unicycler
nano_canu
SRSM4
ill_spades
ill_pac_deduped_combined
ill_nano_deduped_combined
pac_wtdbg2
nano_unicycler
nano_wtdbg2
pac_flye
ill_pac_deduped_combined
nano_canu
pac_canu
ill_nano_deduped_combined
ill_nano_unicycler
ill_spades
pac_wtdbg2
nano_wtdbg2
pac_flye
ill_pac_deduped_combined
nano_unicycler
ill_pac_unicycler
pac_canu
nano_canu
ill_nano_unicycler
ill_spades
pac_wtdbg2
nano_unicycler
pac_flye
nano_wtdbg2
nano_flye
ill_pac_deduped_combined
nano_canu
pac_canu
ill_nano_deduped_combined
ill_spades
ill_pac_unicycler
ill_nano_unicycler
pac_wtdbg2
nano_wtdbg2
pac_flye
ill_pac_deduped_combined
nano_unicycler
ill_pac_unicycler
pac_canu
nano_canu
ill_spades
ill_nano_unicycler
vB_Vpa_sm033
pac_wtdbg2
pac_flye
nano_wtdbg2
nano_unicycler
nano_flye
nano_canu
ill_nano_deduped_combined
ill_pac_deduped_combined
ill_spades
ill_pac_unicycler
ill_nano_unicycler
ill_spades
ill_pac_deduped_combined
ill_nano_deduped_combined
VP1
pac_wtdbg2
pac_flye
nano_unicycler
nano_wtdbg2
nano_flye
nano_canu
ill_nano_deduped_combined
ill_nano_unicycler
ill_pac_unicycler
ill_spades
ill_pac_deduped_combined
1
10
100
1000
Platform
Illumina Only
Nanopore + Illumina
Nanopore Only
PacBio + Illumina
PacBio Only
Indels per 100kb

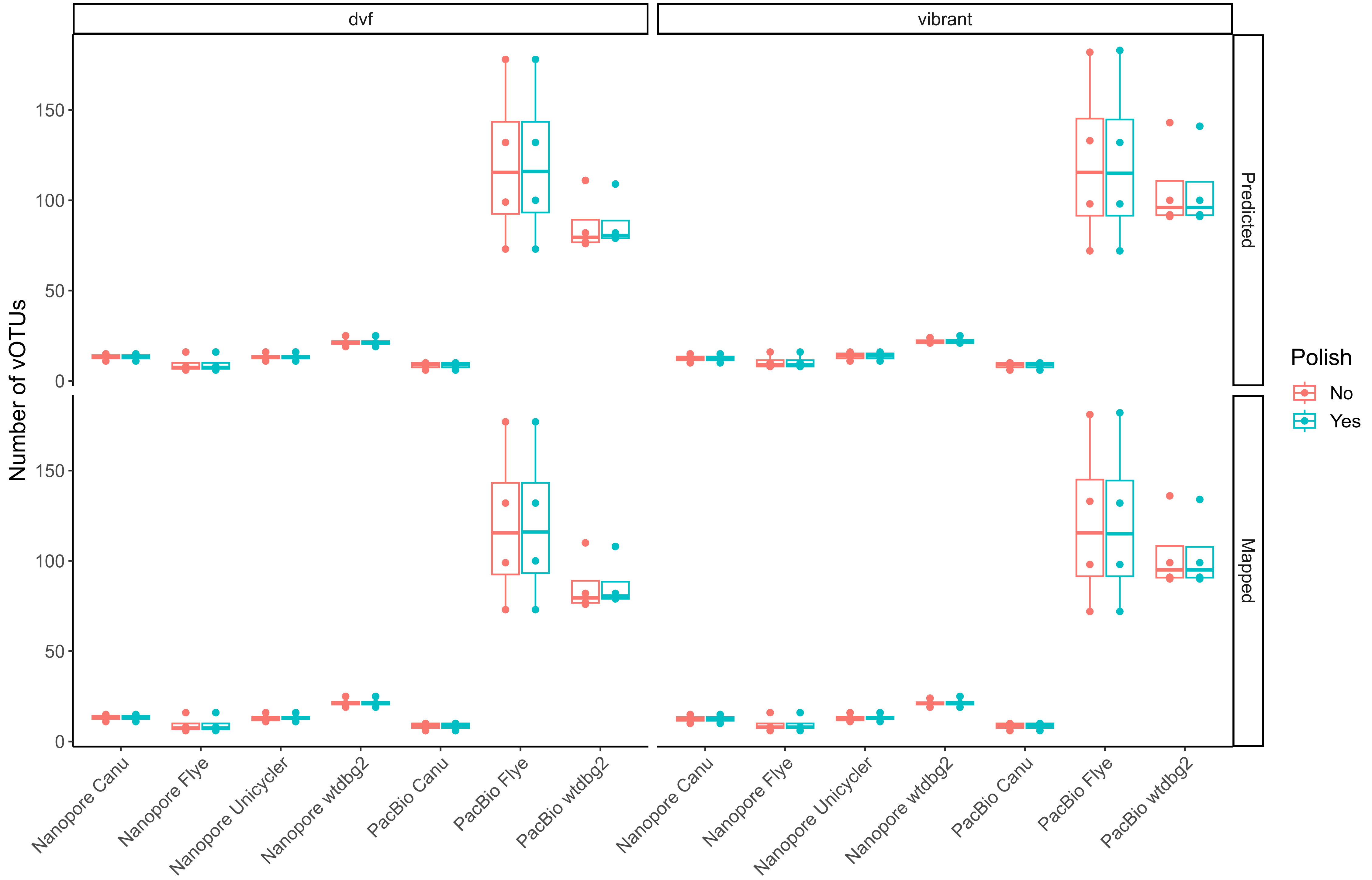

dvf
vibrant
Predicted
Mapped
Number of vOTUs
0
50
100
150
Nanopore Canu
Nanopore Flye
Nanopore Unicycler
Nanopore wtdbg2
PacBio Canu
PacBio Flye
PacBio wtdbg2
Polish
No
Yes

Supplementary Figure 1. Nucleotide similarity of phages in the mock community as determine by ANI from VIRIDIC. Samples with * are not 100% , but as a result of rounding from 99.99%

Supplementary Figure 2. Assembly completeness per genome. Boxplots showing the longest contig aligned to the reference genome as a percentage of genome length. Repeats are for the three separate libraries and additional pooled library.

Supplementary Figure 3. Breaks in Illumina assembly for lowly abundant phages. Plots show molGC% content of (**A**) S-RSM4 and (**B**) vB_VpaS_sm032 plotted against median read depth from the Illumina pooled library. The black circles indicate a break in the assembly.

Supplementary Figure 4. Effect of Medaka polishing on ONT assemblies. (**A**) SNPs and (**B**) INDELs are presented as number per 100 kb of reference genome for each assembly. Results for which at least 50% of the reference genome was recovered by contigs were included. Error bars show standard error of mean and the number above the bar indicates the number of genomes included in mean calculation (from a total possible maximum of 60 (15 genomes, 4 assemblies)).

Supplementary Figure 5. Assembly completeness for long read sequencing per genome. Boxplots showing the NGA50 value as a percentage of reference genome length. Replicates are for the three separate libraries and the additional pooled library.

Supplementary Figure 6. Assembly completeness for long read sequencing per library. Boxplots showing the NGA50 value as a percentage of reference genome length. Individual values correspond to separate genomes.

Supplementary Figure 7. Effect of sequencing technology and assembler on the observed number of SNPs and INDELS. (**A**) SNPs and (**B**) INDELs are presented as number per 100 kb of reference genome for each assembly. Results for which at least 50% of the reference genome was recovered by contigs were included. Error bars show standard error of mean and the number above the bar indicates the number of genomes included in mean calculation (from a total possible maximum of 60 (15 genomes, 4 assemblies)).

Supplementary Figure 8. Effect of sequencing technology and assembler on the observed number of SNPs per genome. SNPs are presented as number per 100 kb of reference genome for each assembly. Results for which at least 50% of the reference genome was recovered by contigs were included.

Supplementary Figure 9. Effect of sequencing technology and assembler on the observed number of INDELs per genome. INDELs are presented as number per 100 kb of reference genome for each assembly. Results for which at least 50% of the reference genome was recovered by contigs were included.

Supplementary Figure 10. The effect of polishing long-read assemblies on viral prediction. Boxplots showing the number of predicted contigs for the five different long-read assemblies

before and after polishing, with the lower two panels showing the number of contigs which mapped to the reference genomes. The left two panels show vOTUs predicted with DeepVirFinder, and the right two panels show predictions from VIBRANT. The individual boxes contain values from three individual libraries and a pooled library.
